# Chaperones, Membrane Trafficking and Signal Transduction Proteins Regulate Zaire Ebola Virus trVLPs and Interact With trVLP Elements

**DOI:** 10.3389/fmicb.2018.02724

**Published:** 2018-11-12

**Authors:** Dong-Shan Yu, Tian-Hao Weng, Chen-Yu Hu, Zhi-Gang Wu, Yan-Hua Li, Lin-Fang Cheng, Nan-Ping Wu, Lan-Juan Li, Hang-Ping Yao

**Affiliations:** ^1^State Key Laboratory for Diagnosis and Treatment of Infectious Diseases, The First Affiliated Hospital, School of Medicine, Zhejiang University, Hangzhou, China; ^2^Collaborative Innovation Center for Diagnosis and Treatment of Infectious Diseases, The First Affiliated Hospital, School of Medicine, Zhejiang University, Hangzhou, China

**Keywords:** Ebola virus life cycle, trVLPs, RNAi screening, glycoprotein, protein 40, immunoprecipitation

## Abstract

Ebolavirus (EBOV) life cycle involves interactions with numerous host factors, but it remains poorly understood, as does pathogenesis. Herein, we synthesized 65 siRNAs targeting host genes mostly connected with aspects of the negative-sense RNA virus life cycle (including viral entry, uncoating, fusion, replication, assembly, and budding). We produced EBOV transcription- and replication-competent virus-like particles (trVLPs) to mimic the EBOV life cycle. After screening host factors associated with the trVLP life cycle, we assessed interactions of host proteins with trVLP glycoprotein (GP), VP40, and RNA by co-immunoprecipitation (Co-IP) and chromatin immunoprecipitation (ChIP). The results demonstrate that RNAi silencing with 11 siRNAs (ANXA5, ARFGAP1, FLT4, GRP78, HSPA1A, HSP90AB1, HSPA8, MAPK11, MEK2, NTRK1, and YWHAZ) decreased the replication efficiency of trVLPs. Co-IP revealed nine candidate host proteins (FLT4, GRP78, HSPA1A, HSP90AB1, HSPA8, MAPK11, MEK2, NTRK1, and YWHAZ) potentially interacting with trVLP GP, and four (ANXA5, GRP78, HSPA1A, and HSP90AB1) potentially interacting with trVLP VP40. Ch-IP identified nine candidate host proteins (ANXA5, ARFGAP1, FLT4, GRP78, HSPA1A, HSP90AB1, MAPK11, MEK2, and NTRK1) interacting with trVLP RNA. This study was based on trVLP and could not replace live ebolavirus entirely; in particular, the interaction between trVLP RNA and host proteins cannot be assumed to be identical in live virus. However, the results provide valuable information for further studies and deepen our understanding of essential host factors involved in the EBOV life cycle.

## Introduction

Ebolavirus (EBOV) is a single-stranded, negative-sense RNA virus with a heterogeneous filamentous structure (Jun et al., 2015) that was first reported in 1976 as the cause of Ebola viral disease (EVD) in humans and other primates (Johnson et al., 1977). Five different EBOVs have been defined: Ebola virus (EBOV, previously known as Zaire ebolavirus), Sudan virus (SUDV), Bundibugyo virus (BDBV), Taï Forest virus (TAFV) and Reston virus (RESTV) (Kuhn, 2017). The EVD epidemic from December 2013 to March 2016 in Western Africa was caused by EBOV, and was associated with >28,000 cases and >11,000 deaths in 11 countries (Ajisegiri et al., 2018).

Although numerous studies have been devoted to EVD therapeutics, such as immune-based treatments and small molecule inhibitors, and considerable progress has been made (Fabozzi et al., 2011; Oestereich et al., 2014; Wolf et al., 2015; Sissoko et al., 2016), the molecular basis of the EBOV life cycle and its relevance to pathogenesis remain poorly understood. For example, the mechanisms, proteins and receptors mediating viral entry, the host enzymes triggering and accelerating uncoating and virus fusion, and the host factors facilitating VP40 oligomerisation and association of VP40 with the inner leaflet remain largely unknown (Johnson et al., 2016; Gc et al., 2017; Younan et al., 2017; Kurosaki et al., 2018).

To model the EBOV life cycle using a safe method, a series of trVLPs have been developed (Hoenen et al., 2014). These trVLPs could express the EBOV proteins required for genome replication and transcription to model EBOV life cycles under biosafety level 2 conditions. Based on the EBOV life cycle in host cells, and on previous RNAi screening that identified filovirion-associated and secretory pathway-related host factors (Spurgers et al., 2010; Simpson et al., 2012; Sakurai et al., 2015), we herein synthesized siRNAs targeting host genes connected to membrane traffic machinery, endoplasmic reticulum-Golgi recycling, inhibition of secretion, and lipid droplet formation, and assessed their role in the EBOV life cycle. These genes are mostly connected with virus life cycle, specifically viral entry, uncoating, fusion, replication, assembly and budding.

In the EBOV life cycle, GP is critical for EBOV entry, uncoating and fusion processes (Nanbo et al., 2010; Takada, 2012), while VP40 also plays a crucial role in EBOV assembly and budding (Dessen et al., 2000; Balmith and Soliman, 2017), and both proteins engage in important interactions with host cellular and plasma membranes. Thus, host factors that exert a significant influence on the EBOV life cycle were identified by RNAi silencing, and interactions between EBOV GP, VP40, and nucleic acids were evaluated by Co-IP or ChIP. This is the first systematic survey of host factors that affect the EBOV life cycle, demonstrating interactions between host proteins and EBOV GP, VP40 and RNA.

## Materials and Methods

### Cell Line and Plasmids

Human embryonic kidney (HEK) 293T cells were cultured in DMEM (Thermo Fisher, Waltham, MA, United States; Cat# 10566016) containing 10% FBS (Gibco, Waltham, MA, United States; Cat# 10099141), 2 mM L-glutamine (Life Technologies, Waltham, MA, United States; Cat# 25030081), and 1% penicillin- streptomycin (Life Technologies; Cat# 10378016) at 37°C with 5% CO_2_. Plasmids pCAGGS-VP30, pCAGGS-VP35, pCAGGS-NP, pCAGGS-L, p4cis-vRNA-RLuc, pCAGGS-Tim1, and pCAGGS-T7 were kindly provided by Drs. Heinz Feldmann and Thomas Hoenen, Rocky Mountain Laboratories, National Institute of Health (NIH). Plasmid p4cis-vRNA-RLuc containing EBOV non-coding regions, a reporter gene, and three genes (VP40, GP1, 2 and VP24) involved in morphogenesis, budding and cell entry, was used to produce trVLPs. In the following experiments, all tests were carried out with EBOV trVLPs.

### Production of trVLPs

Producer 293T cells (p0) in 6-well plates were transfected with plasmids encoding each EBOV structural protein (75 ng pCAGGS-VP30, 125 ng pCAGGS-VP35, 125 ng pCAGGS-NP, 1,000 ng pCAGGS-L, 250 ng p4cis-vRNA-RLuc), and pCAGGS-T7 (250 ng) encoding a Renilla luciferase reporter (Hoenen et al., 2014). Cell supernatants (200 ml) from 10 6-well plates containing released trVLPs were harvested at 72 h post-transfection, and cells and cellular debris were pelleted by gentle centrifugation at 175 × *g*. The supernatant was then used to infect target 293T cells (p1) previously transfected with RNP components (125 ng pCAGGS-NP, 125 ng pCAGGS-VP35, 75 ng pCAGGS-VP30, 1,000 ng pCAGGS-L, 250 ng pCAGGS-Tim1) (Hoenen et al., 2014). Target 293T cells (p2–p5) were treated in a similar manner. Cleared supernatants (33 ml) in 2 ml of 20% sucrose from the bottom of each tube were centrifuged at 125,000 × *g* in a SW-28 rotor for 3 h at 4°C. The resulting pellets were resuspended in 100 μl ice-cold NTE buffer (10 mM Tris pH 7.5, 100 mM NaCl, 1 mM EDTA) by tapping gently about 100 times, and trVLPs were stored on ice or in a refrigerator at 4°C until use.

### Determining the 50% Tissue Culture Infective Dose (TCID50) of trVLPs

To determine the TCID50 values of trVLPs, CPEs were observed using microscopy, and the results from each dilution were used to calculate TCID50 values using the Reed–Muench method (Ramakrishnan, 2016). After harvest, trVLPs were 10-fold serially diluted with DMEM at concentrations ranging from 10^-1^ to 10^-10^. Attenuated trVLPs (100 μl) were added to eight wells in each row of a 96-well plate, and 293T cell suspension (100 μl) was added to each well to a final cell density of 2 × 10^5^ cells/ml. Additionally, 293T cells not infected with trVLPs were included as controls. CPEs were observed and recorded each day for 7 days, and TCID50 values were calculated according the Reed–Muench method, giving a value of 10^3.76^/0.1 ml (detailed data are listed in Supplementary Table S1).

### Electron Microscopy (EM) Analysis of trVLPs

Purified trVLPs were pipetted onto a 300-mesh copper grid coated with carbon film, incubated for 5 min at room temperature, and grids were washed twice with distilled water and negatively stained for 15 s with 1% uranyl acetate. Excess liquid was removed with a filter paper and trVLPs were visualized under a Hitachi H7000 transmission electron microscope.

### Imaging of DiI-Labeled trVLP Internalization in Live 293T Cells

Purified trVLPs (∼100 μg viral protein) were resuspended in NTE buffer a final volume of 1 ml and 1,1′-dioctadecyl-3,3,3′,3′-tetramethylindocarbocyanine perchlorate (DiI, dissolved in ethanol to a final concentration of 10 μM; Invitrogen, Waltham, MA, United States; Cat# D282) was added and mixed thoroughly. The mixture was shaken gently for 1 h at room temperature then passed through a 0.22 μm filter (Millipore, Darmstadt, Germany; Cat# SLGP033RB). HEK 293T cells cultured in 8-well chamber slides with removable wells were mixed with 4′,6-diamidino-2-phenylindole (DAPI; Invitrogen; Cat# P36931) at 10 μg/ml for 10 min to stain nuclei, and 293T cells were then incubated with DiI-labeled trVLPs at 4°C for 10 min and washed with PBS. The internalization of DiI-labeled trVLPs in 293T cells was imaged at different times from 10 to 60 min at 37°C on the stage of a fluorescence microscope.

### Selection of Candidate Genes

To select host factors that are potentially related to various stages of the EBOV life cycle (including viral entry, uncoating, fusion, replication, assembly and budding), we chose host genes that are associated with membrane traffic machinery, endoplasmic reticulum-Golgi recycling, inhibition of secretion, and lipid droplet formation. For example, the knockdown of FLT4, RAB11B, and PLCH1 resulted in high-level secretion inhibition; GOSR1, LTK, and CNKSR1 are linked to endoplasmic reticulum-Golgi recycling; VAMP2, RAB11B, and COPG are membrane traffic machinery-related molecules (Simpson et al., 2012); HSPA8, HSP90AB1, and ANXA5 are believed to be related to filamentous virus (Spurgers et al., 2010). A total of 65 genes probably associated with the EBOV life cycle or virus life cycle-related functions (Spurgers et al., 2010; Simpson et al., 2012; Sakurai et al., 2015) were selected according to preliminary screening. Details and references for these genes are listed in Supplementary Table S3.

### SiRNA Screening and trVLP Infection

SiRNAs targeting the 65 selected genes were designed and three sequences were synthesized for each gene. To choose the most efficient sequence for RNA interfere (RNAi), the gene knockdown efficiency of each sequence was evaluated by quantitative PCR, with the 18sRNA gene as a control. Values were calculated using the 2^-ΔΔ^*^C^*^t^ method, and the smaller the value, the higher the knockdown efficiency. The most efficient siRNAs were chosen for RNAi screening tests (data listed in Supplementary Table S2), and details of each chosen siRNA are included in Supplementary Table S3. In 24-well plates, 100 μl of opti-MEM medium (Invitrogen; Cat# 31985070) containing 1.4 μl siRNA and 4.5 μl HiPerFect transfection reagent (Qiagen, Dusseldorf, Germany; Cat# 301705) was added to each well and plates were shaken gently for 1 min. After 10 min incubation at room temperature, a cell suspension (400 μl) containing 1 × 10^5^ cells was added to give a final siRNA concentration of 75 nM. Cells were incubated at 37°C and 5% CO_2_ for 48 h, washed with PBS, and infected with trVLPs (TCID50 = 10^2.76^/0.01 ml) for 48 h. Negative control siRNA (Qiagen; Cat# 1027310) served as a control.

### RNA Extraction and Quantitative Real-Time Reverse Transcription PCR (qRT-PCR)

After trVLP infection, 293T cells in 24-well plates were washed with PBS, and total RNA was extracted from cells using TRIzol (Invitrogen; Cat# 15596018) according to the manufacturer’s instructions. qRT-PCR was performed with a EBOV nucleic acid test kit (Zhijiang Bio-tech, Shanghai, China; Cat# QR-0220-02) on an ABI 7500 qPCR system (45°C for 10 min and 95°C for 15 min, followed by 45 cycles at 95°C for 15 s and 60°C for 30 s). The positive control, provided in the kit, was serially diluted over three orders of magnitude, and the absolute quantity of EBOV RNA (copy/ml) was calculated using the comparative Ct method and a standard curve. All qRT-PCR experiments were performed in triplicate and repeated independently three times.

### Antibodies

Antibodies recognizing HSP70 (HSPA1A; Cat# 4873S), HSP90β (HSP90AB1; Cat# 5087S), MEK1/2 (Cat# 9122S), ARFGAP1 (Cat# 14522S) and Trk (NTRK1; Cat# 92991S) were purchased from Cell Signaling Technology (Danvers, MA, United States). Antibodies recognizing MAPK11 (Cat# ab183208), VEGF Receptor 3 (FLT4; Cat# ab10284), Annexin V (ANXA5; Cat# ab54775), GRP78 (HSPA5; Cat# ab21685), GAPDH (Cat# ab8245), and Ebola virus GP (Cat# ab1918) were purchased from Abcam (Cambridge, United Kingdom). Antibodies recognizing 14-3-3 zeta/beta (YWHAZ; Cat# NB100-1964) and Hsc70 (HSPA8; Cat# NBP2-12880) were purchased from Novus Biologicals (Littleton, CO, United States). Antibody recognizing Ebola virus VP40 (Cat# sc-51872) was purchased from Santa Cruz Biotechnology (Dallas, TX, United States). Antibodies against HSPA1A, HSP90AB1, MEK1/2, ARFGAP1, NTRK1, MAPK11, FLT4, and GRP78 were rabbit-derived IgGs. Antibodies against ANXA5, YWHAZ, and HSPA8 were mouse-derived IgGs. Normal mouse IgG (Cat# SC-2025) and normal rabbit IgG (Cat# SC-2027) were obtained from Santa Cruz Biotechnology and used as isotype controls in immunoprecipitation tests.

### Co-IP and Immunoblot Analysis

HEK 293T cells in 12-well plates were infected with trVLPs (TCID50 = 10^3.76^/0.1 ml) for 48 h and whole-cell extracts were prepared followed by incubation overnight with appropriate antibodies recognizing host proteins plus Protein G beads (GE Healthcare, Uppsala, Sweden; Cat# 28-9440-08). Antibodies mixed with Protein G beads and normal cell/extracts without trVLPs, and Protein G beads mixed with trVLP-treated cell extracts without antibodies, were included as control groups. Beads were then washed five times with low-salt lysis buffer, and immunoprecipitates were eluted with 2× SDS Loading Buffer and resolved by SDS-PAGE. Proteins were transferred to PVDF membranes and further incubated with anti-GP or VP40 antibodies. Proteins of interest were detected with the Super Signal West Pico chemiluminescent substrate (Thermo Fisher; Cat# 34580). To confirm that siRNAs interfered with the expression of target proteins in a specific manner, immunoblot analysis of target host proteins was performed after siRNA transfection. Meanwhile, to confirm the specificity of the antibodies, target host proteins, GP, and VP40 proteins from the above extracts were tested before and after Co-IP separately (normal mouse or rabbit IgG served as isotype controls). The housekeeping protein GAPDH was used as an internal control for immunoblot analyses.

### Chromatin Immunoprecipitation (ChIP)

After infection of trVLPs (TCID50 = 10^2.76^/0.01 ml) for 48 h, HEK 293T cells in 12-well plates were fixed with 1% formaldehyde for 10 min, and glycine (Thermo Fisher; Cat# 28363) was added to terminate the cross-linking reaction. Whole-cell extracts were then prepared using a Simple ChIP Enzymatic Chromatin IP Kit (Cell Signaling Technology; Cat# 9003) following the manufacturer’s instructions with appropriate antibodies recognizing host proteins, and normal mouse or rabbit IgGs served as isotype controls. Beads were then washed and cross-linking substances were unlocked according the ChIP kit instructions. Purified nucleic acids were then amplified by qPCR with a qPCR Probe Kit (Vazyme Biotech, Nanjing, China; Cat# Q2223-01). Primers and probe were designed according to the sequence of *Zaire ebolavirus* isolate *Homo sapiens*-wt/GIN/2014/Gueckedou-C05 (GenBank No. KJ660348.2) as follows: EN-Z-F, ATGATGGARGCTACGGCG; EN-Z-R, TGCCCTCTGTATGCTGGCCCT; EN-Z-P, CCARAGTTACTCGGAAAACGGCATG.

### Sequencing Analysis

The PCR products amplified from ChIP samples were purified by agarose gel electrophoresis, recycled, and sequenced by the Sanger chain termination method. Genotypes were attributed using BioEdit software (Ibis Biosciences, Carlsbad, CA, United States) for allelic discrimination.

### Statistical Analysis

Data in normal distributions are presented as the mean ± standard deviation (Kim et al., 2018). Student’s *t*-tests (two-tailed, paired or unpaired *t*-tests with Welch’s correction), and multiple comparisons were performed where appropriate. All analysis was performed with Graphpad Prism 5 (GraphPad Software, United Kingdom) and differences between group means with *p* < 0.05 were considered statistically significant.

## Results

### EBOV-trVLPs Successfully Simulate Authentic Ebola Virions

**FIGURE 1:**
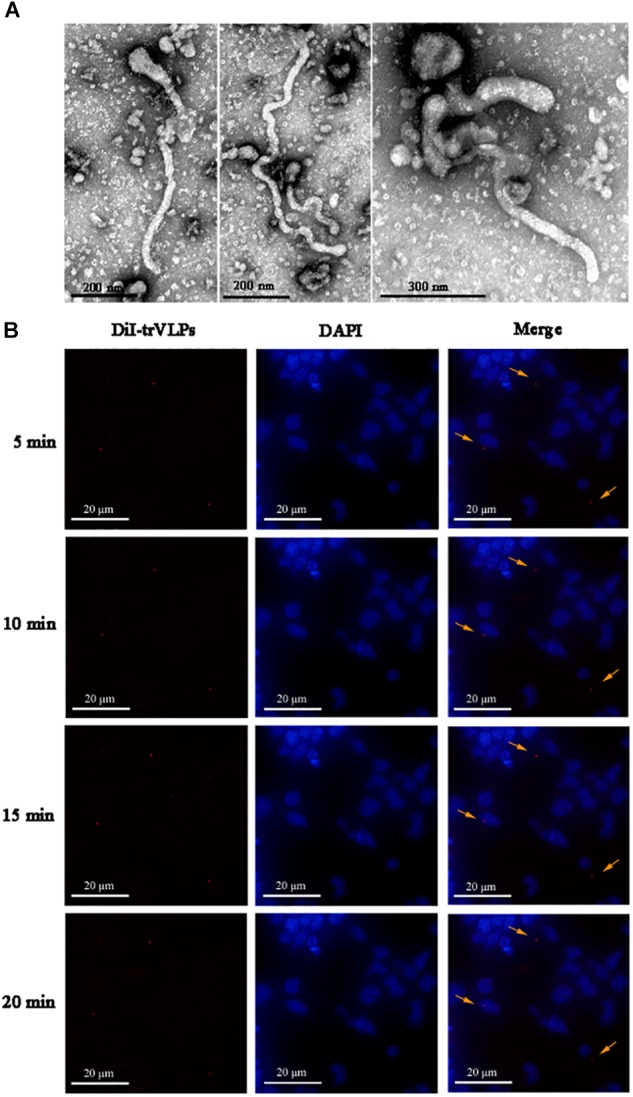
Visualization and identification of ebolavirus transcription- and replication-competent virus-like particle (EBOV-trVLP). **(A)** Visualization of ebolavirus (EBOV) transcription- and replication-competent virus-like particles (trVLPs) by electron microscopy (EM). trVLPs were purified by ultracentrifugation using a 20% sucrose gradient and particles were visualized by EM after negative staining. The arrow indicates filamentous-like viral particles. **(B)** Internalization and tracing of 1,1′-dioctadecyl-3,3,3′,3′-tetramethylindocarbocyanine perchlorate (DiI)-labeled trVLPs at 5, 10, 15, and 20 min after infecting 293T cells by fluorescence microscopy (indicated by yellow arrows).

EBOV-trVLPs synthesized as described above displayed a filamentous-like morphology with a diameter of 100–500 μm as visualized by EM (Figure 1A). Meanwhile, internalization and tracking of DiI-labeled trVLPs in live 293T cells visualized by fluorescent microscopy indicated that trVLPs gradually translocated into the cytoplasm over time (Figure 1B). It has been reported that filamentous viruses, including authentic EBOV, are internalized into cells following stimulation of the macropinocytosis pathway, while spherical pseudotype virions are internalized via the endocytosis pathway (Nanbo et al., 2010). In our present study, trVLPs are filamentous virions, hence we presumed that trVLPs would be internalized via the macropinocytosis pathway.

### SiRNA Knockdown of Host Factors Decreases Replication of trVLPs

**FIGURE 2:**
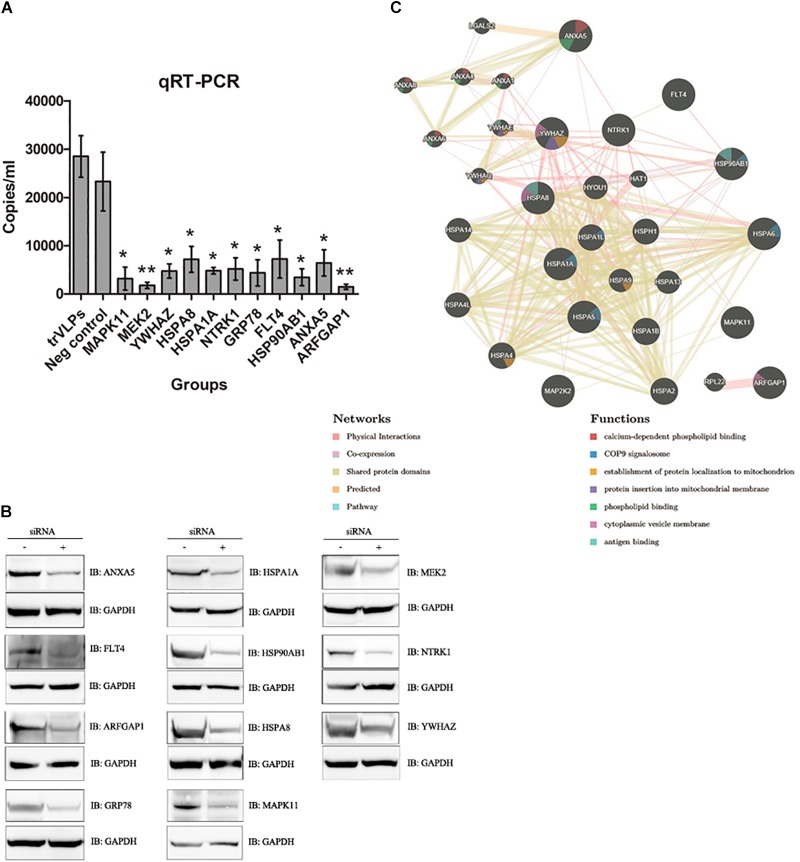
RNAi screening of host factors related to EBOV-trVLP life cycle and further validation and analysis. **(A)** RNAi silencing analysis of host factors required for the EBOV trVLP life cycle. A 100 μl sample of opti-MEM medium containing 1.4 μl siRNA and 4.5 μl HiPerFect was placed in 24-well plates, and a cell suspension (400 μl) containing 1 × 10^5^ cells was added to give a final siRNA concentration of 75 nM. After incubation for 48 h, cells were infected with trVLPs for another 48 h, total RNA was extracted, and the absolute quantity of EBOV RNA was measured using a EBOV nucleic acid test kit. All qRT-PCR experiments were performed in triplicate and repeated three times independently. Cells not transfected with siRNA but infected with trVLPs served as a blank control; cells transfected with isotype siRNA and infected with trVLPs served as a negative control. Eleven siRNAs targeting HSPA1A, HSP90AB1, ARFGAP1, ANXA5, YWHAZ, MAPK11, NTRK1, FLT4, GRP78, MEK2, and HSPA8 inhibited trVLP replication effectively. ^∗^*p* < 0.05, ^∗∗^*p* < 0.01. **(B)** Western blot analysis of each target protein after siRNA transfection. The 11 target proteins were all expressed at much lower levels following transfection with the relevant siRNA. Normal 293T cells and cells transfected with isotype siRNAs served as controls. **(C)** Characterization of selected networks of candidate host proteins that may be important for regulating the trVLP life cycle. Interactions of the 11 host proteins were assessed using the GeneMANIA interaction database, and their functions were evaluated in association with cytoplasmic vesicle membranes, mitochondrial membranes, mitochondria, antigen binding, the COP9 signalosome, and phospholipid binding.

To identify factors associated with the life cycle of trVLPs, siRNAs targeting 65 selected genes were tested in 293T cells infected with trVLPs in triplicate by qRT-PCR. The results showed that siRNA knockdown of ANXA5, ARFGAP1, FLT4, GRP78, HSPA1A, HSP90AB1, HSPA8, MAPK11, MEK2, NTRK1, and YWHAZ efficiently inhibited trVLP replication (*p* < 0.05; Figure 2A). To confirm that these siRNAs interfered with the expression of target proteins in a specific manner, we measured the levels of target proteins by western blot analysis, and this demonstrated significant interference for each target protein as expected (Figure 2B). The results suggest that these host proteins are highly likely to participate in the trVLP life cycle, and may perform critical roles. In addition, the results from the siRNA knockdowns of other host proteins suggest that they do not interfere with trVLP replication (Supplementary Figure S1).

Among these selected candidate host factors, four are chaperones (HSPA1A, HSP90AB1, HSPA8, and GRP78), four are involved in plasma and/or endosome membrane trafficking (ANXA5, ARFGAP1, FLT4, and NTRK1), and three play important roles in signal transduction pathways (MAPK11, MEK2, and YWHAZ). Further bioinformatic analysis by GeneMANIA revealed that these candidate host factors were markedly enriched in gene categories associated with 14-3-3 protein families [such as YWHAZ, YWHAE and YWHAQ)], annexin families (including ANXA1, ANXA4, and ANXA5), heat shock protein (HSP) families (e.g., HSPA1A, HSP90AB1, GRP78, and HSPA8), and MAP kinase families (including MAPK11 and MAP2K2; Figure 2C). The analysis also indicated that candidate host factors are associated with cytoplasmic vesicle membranes, mitochondrial membranes, mitochondria, the COP9 signalosome, and phospholipid binding, and strongly connected with antigen binding, viral endocytosis, transportation, anti-virus responses, and viral budding.

Since GP and VP40 proteins perform critical functions in the EBOV life cycle and since both interact with host cell membranes, we performed Co-IP and Ch-IP assays to validate host protein-GP/VP40 and host protein-trVLP nucleic acid interactions.

### Candidate Host Proteins Interact With trVLP GP

**FIGURE 3:**
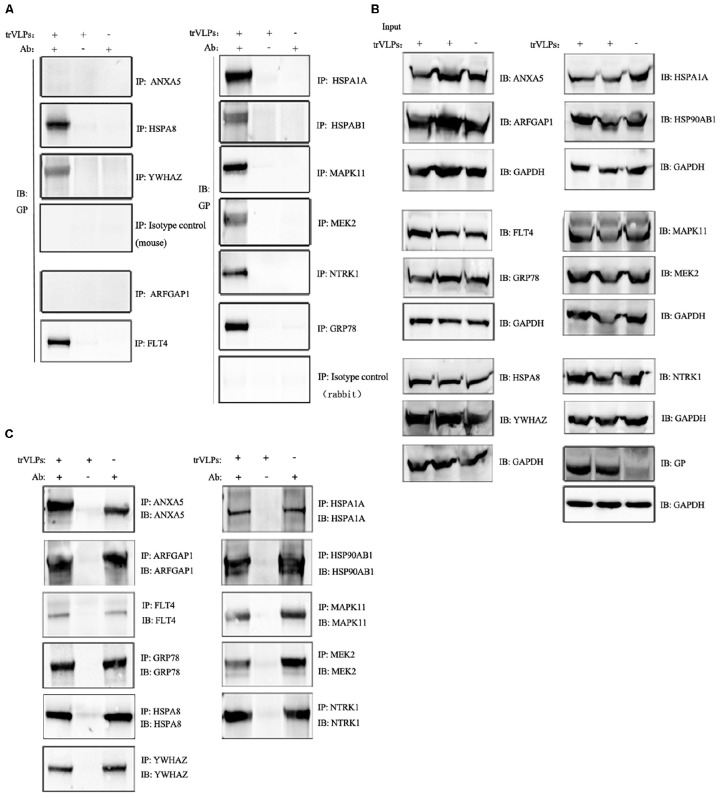
Co-immunoprecipitation (Co-IP) and immunoblot analysis of interaction between target proteins and EBOV-trVLP glycoprotein (GP). **(A)** Co-IP and immunoblot analysis of EBOV trVLP GP. Extracts from HEK 293T cells infected with trVLPs were incubated with antibodies recognizing candidate host proteins plus Protein G beads; pulled-down proteins were detected by western blotting using anti-GP antibody. HSPA1A, MAPK11, NTRK1, GRP78, FLT4, HSPA8, MEK2, HSP90AB1, and YWHAZ interact with GP, whereas ARFGAP1, ANXA5, and isotype controls do not. Extracts incubated with Protein G beads without antibody, and antibodies mixed with Protein G beads and normal cell extracts without trVLPs served as controls. **(B)** Immunoblot analysis of the 11 target proteins before IP and **(C)** after IP. Samples were prepared as described above.

Antibodies recognizing the 11 host proteins were incubated overnight with Protein G beads and cell extracts infected with trVLPs described above. Immunoblot analysis was then carried out with anti-GP antibody, and the results demonstrated that nine host proteins (FLT4, GRP78, HSPA1A, HSP90AB1, HSPA8, MAPK11, MEK2, NTRK1, and YWHAZ) interacted with GP, whereas ARFGAP1 and ANXA5 did not appear to interact with GP (normal mouse or rabbit IgG served as isotype controls; Figure 3A). Meanwhile, immunoblot analyses of the 11 target host proteins before and after IP indicated a high degree of specificity for those antibodies, further verifying the interactions between GP and candidate host proteins (Figures 3B,C).

### Candidate Host Proteins Interact With trVLP VP40

**FIGURE 4:**
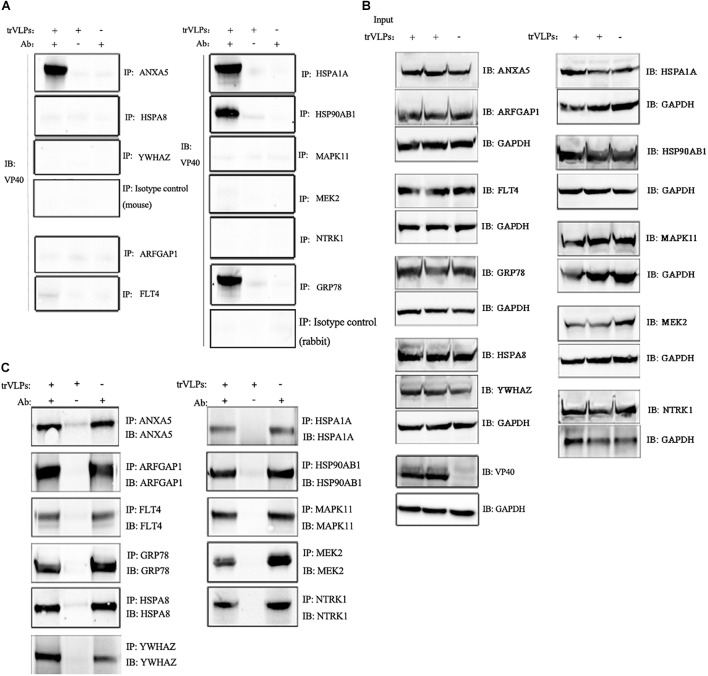
Co-immunoprecipitation and immunoblot analysis of interaction between target proteins and EBOV-trVLP VP40. **(A)** Co-IP and immunoblot analysis of EBOV trVLP protein 40 (VP40). Extracts from HEK 293T cells infected with trVLPs were incubated with antibodies recognizing candidate host proteins plus Protein G beads, and pulled-down proteins were detected by western blotting using anti-VP40 antibody. HSP90AB1, ANXA5, HSPA1A, and GRP78 interact with VP40, whereas ARFGAP1, YWHAZ, MAPK11, NTRK1, FLT4, MEK2, HSPA8, and isotype controls do not. Extracts incubated with Protein G beads without antibody, and antibodies mixed with Protein G beads and normal cell extracts without trVLPs served as controls. **(B)** Immunoblot analysis of the 11 target proteins before IP and **(C)** after IP. Samples were treated as described above.

Next, interactions of the 11 candidate host proteins with VP40 were tested by incubating antibodies recognizing the 11 host proteins overnight with Protein G beads and trVLPs-treated cell extracts. Western blotting with anti-VP40 antibody indicated that ANXA5, GRP78, HSPA1A, and HSP90AB1 interacted with VP40, whereas ARFGAP1, HSPA8, MAPK11, MEK2, FLT4, NTRK1, and YWHAZ did not interact with VP40 (normal mouse or rabbit IgG served as isotype controls; Figure 4A). Immunoblot analyses of the 11 candidate host proteins before and after IP confirmed the specificity of those antibodies, as well as interactions between VP40 and candidate host proteins (Figures 4B,C).

### Candidate Host Proteins Interact With trVLP RNA

**FIGURE 5:**
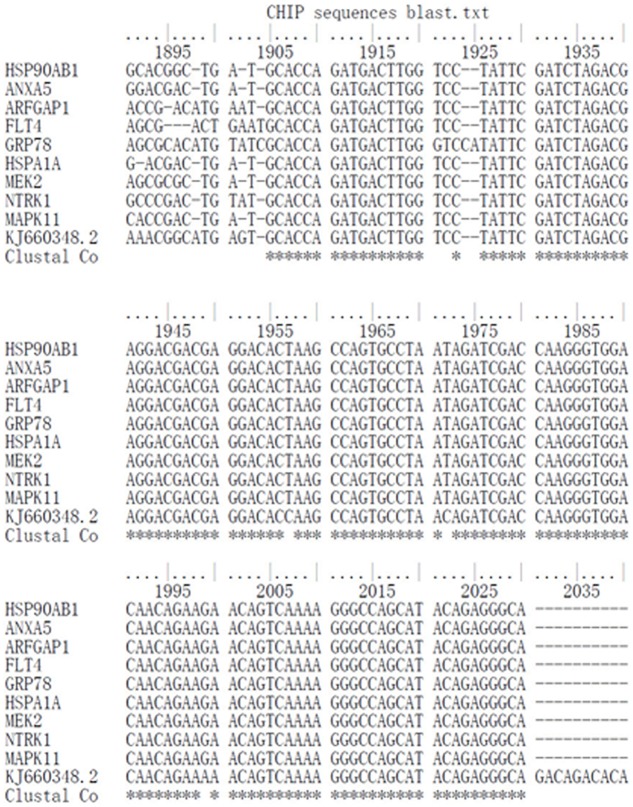
Chromatin immunoprecipitation (ChIP) and DNA sequencing analysis of EBOV trVLP nucleic acids. Extracts from HEK 293T cells infected with trVLPs were processed and pulled down using a Chromatin IP Kit with antibodies recognizing candidate host proteins. Purified nucleic acids were amplified by qPCR, and products were sequenced by the Sanger chain termination method. Genotypes were attributed and compared with the Zaire ebolavirus sequence (GenBank No. KJ660348.2) using BioEdit software. Samples from HSP90AB1, ANXA5, ARFGAP1, FLT4, GRP78, HSPA1A, MEK2, NTRK1, and MAPK11 are consistent with sequences from EBOV, while HSPA8 and YWHAZ are not. Isotype negative controls were included.

Since interactions between virus RNA and host proteins are likely to be essential for viral replication, we next investigated whether trVLP RNA interacts with the candidate host proteins using Ch-IP assays and purified nucleic acids by qPCR and DNA sequencing. BLAST analysis of the sequencing results indicated that sequences from nine antibodies recognizing candidate host proteins (HSP90AB1, ANXA5, ARFGAP1, FLT4, GRP78, HSPA1A, MEK2, NTRK1, and MAPK11) were consistent with *Zaire ebolavirus* (GenBank No. KJ660348.2; Figure 5). HSPA8, YWHAZ, and isotype controls did not recognize trVLP RNA.

## Discussion

A better understanding of the mechanisms of the EBOV life cycle and host–virus interactions (including protein–protein and protein–RNA interactions) would greatly assist the elucidation of EBOV pathogenesis and therapeutic development. Herein, we identified 11 candidate host proteins by RNAi silencing that appear to function in the EBOV-trVLP life cycle among 65 host factors presumed to be associated with the virus life cycle and secretary pathways. Meanwhile, we measured candidate host protein levels after RNAi by western blot analysis, and the results revealed significant interference effects and confirmed the specificity of RNAi silencing. Bioinformatic analysis by GeneMANIA indicated that these candidate host proteins belong to the following families: (1) HSPs that participate in a wide variety of cellular processes, including activation of proteolysis, protecting the proteome against stress, infection, and transport of newly synthesized polypeptides (Alvarez-Sanchez et al., 2015; Chaudhary et al., 2016; Heck et al., 2017); (2) 14-3-3 protein families that bind a number of signaling proteins including kinases, phosphatases and transmembrane receptors (Radhakrishnan et al., 2012; Obsilova et al., 2014; Aghazadeh and Papadopoulos, 2016); (3) Annexin families that are involved in trafficking and organization of endocytosis, vesicles, exocytosis, and calcium ion channel formation (Grieve et al., 2012; Kuehnl et al., 2016); (4) MAP kinase families, which play critical roles in signal transduction, phosphorylation and activation of MAPK1/ERK2 and MAPK3/ERK1 pathways (Vomastek et al., 2008; Robinson and Pitcher, 2013; Cook et al., 2017). Further functional analysis confirmed that the candidate host proteins were strongly associated with cytoplasmic vesicle membranes, mitochondrial membranes, mitochondria, the COP9 signalosome, and phospholipid binding, and connected with antigen binding, viral endocytosis, transportation, anti-virus responses, and viral budding (Bech-Otschir et al., 2001; Horner et al., 2011; van Zuylen et al., 2012; Richard et al., 2015).

Heat shock protein family proteins are known to be associated with many viruses. For example, the Hsp70 chaperone network mediates the dengue virus life cycle, GRP78 is a potential defensive molecule against hepatitis A virus replication, and Hsp90 controls reactivation of human immunodeficiency virus type 1 from latency (Anderson et al., 2014; Taguwa et al., 2015; Vashist et al., 2015; Jiang et al., 2017). Meanwhile, filovirion studies showed that HSP70 family proteins are associated with Ebola virus (Nelson et al., 2017), consistent with our current results. Members of 14-3-3 protein families are also connected with viral infection, since they mediate signaling pathways and interactions during viral dsRNA stimulation and advanced antiviral innate immune responses (Ohman et al., 2010; Ohman et al., 2014). However, interactions of 14-3-3 proteins with EBOV have not been reported previously. Similarly, annexin family proteins also interact closely with RNA viruses such as measles virus and H5N1 avian influenza virus (Ma et al., 2017; Koga et al., 2018), but interactions with EBOV have not been reported previously. MAP kinases are essential in signal transduction and involved in numerous virus life cycle events, including in EBOV (Strong et al., 2008; Johnson et al., 2014), but the detailed mechanisms of interactions between MAP kinases and EBOV remain ambiguous.

To confirm the interactions of candidate host proteins with EBOV, Co-IP, and Ch-IP were performed. The results revealed that HSPA1A, HSP90AB1, HSPA8, MAPK11, NTRK1, MEK2, FLT4, GRP78, and YWHAZ interacted with EBOV GP. Meanwhile, immunoblot analyses of the 11 target host proteins before and after IP confirmed the high degree of specificity for those antibodies, and specific interactions between GP and candidate host proteins. Four of these proteins belong to HSP families, which are involved in a variety of cellular functions, and some of the others function in plasma and/or endosome membrane trafficking and signal transduction pathways. These results are consistent with the functions of EBOV GP, which is critical for EBOV cell entry, and uncoating and fusion processes occurring at the cell membrane through the endosomal pathway. Protein interactions can involve various mechanisms including subunit polymerization, cross polymerization, and molecular recognition. However, the detailed mechanisms of the interactions between GP and host proteins remain unclear. Meanwhile, it is worth noting that additional factors likely act as a bridge between the candidate host proteins and GP.

Co-immunoprecipitation and immunoblot analyses also revealed that ANXA5, GRP78, HSPA1A, and HSP90AB1 interact with VP40, and bioinformatic analysis indicated that ANXA5 engages in phospholipid binding, while GRP78 and HSP90AB1 are associated with the COP9 signalosome, which is believed to act as a scaffold to facilitate spatial sequestration for multiple interacting molecules that regulate protein stability, transcription, protein phosphorylation and intracellular distribution (Lee et al., 2013; Mosadeghi et al., 2016). The functions of these candidate host proteins are consistent with the role of VP40 in the EBOV life cycle, which presides over association with plasma membrane phosphatidylserine, the inner leaflet, and translocation of virions through the plasma membrane (Dessen et al., 2000; Balmith and Soliman, 2017). Regardless of whether the modes of interaction between the candidate host proteins and VP40 are direct, indirect, or complex, these proteins appear to be linked to the EBOV life cycle.

Interactions of host proteins with trVLP RNA were assessed, and the results indicated that HSP90AB1, HSPA1A, GRP78, ANXA5, ARFGAP1, FLT4, MAPK11, MEK2, and NTRK1 interact with trVLP RNA. Protein-RNA interactions are important for various cellular process including transcriptional and post-transcriptional regulation, and activating immune responses against virus infection (Bidet and Garcia-Blanco, 2014; Cheng et al., 2015; Borodavka et al., 2017; Cipriano and Ballarino, 2018). In the EBOV life cycle, viral nucleic acids are exposed after uncoating in the late endosome, and negative-sense RNA then acts as a template for the synthesis of positive-sense RNA, which guides transcription of viral mRNA. Host protein-virus RNA interactions are presumably crucial during the EBOV life cycle, as indicated by our results.

There were some limitations to our study. Firstly, all the experiments were based on EBOV trVLPs and HEK 293T cells, the interactions may not reflect the physiology of live EBOV. Further experiments using EBOV are therefore required to confirm these current findings. Secondly, the interaction patterns are unclear, which may work either through direct binding or through the indirect action of one or more intermediate molecules, or through the formation of protein complexes by bridge proteins. Furthermore, it is unclear which region or segment of the GP or VP40 protein interacts with the host proteins. Finally, as ebolavirus probably infects different cell types via different mechanisms, the interactions we found in 293T cells may not be relevant in other host cells, such as monocytes and hepatocytes (Kindrachuk et al., 2014; Rogers and Maury, 2018). Subsequent experiments will attempt to define the patterns of interactions, clarify the specificity and binding sites.

## Conclusion

Analysis of EBOV life cycle-associated host factors by RNAi silencing identified four chaperones (HSPA1A, HSP90AB1, HSPA8, and GRP78), four plasma and endosome membrane trafficking molecules (ANXA5, ARFGAP1, FLT4, and NTRK1) and three signal transduction elements (MAPK11, MEK2, and YWHAZ) that may be essential for EBOV replication. Among these host proteins, nine (FLT4, GRP78, HSPA1A, HSP90AB1, HSPA8, MAPK11, MEK2, NTRK1, and YWHAZ) potentially interact with trVLP GP, four (ANXA5, GRP78, HSPA1A, and HSP90AB1) potentially interact with trVLP VP40, and nine (ANXA5, ARFGAP1, FLT4, GRP78, HSPA1A, HSP90AB1, MAPK11, MEK2, and NTRK1) participate in host protein-trVLP RNA interactions. Although EBOV trVLP cannot replace live ebolavirus entirely, and these interactions cannot be assumed to be identical in live virus, the study provides valuable foundations for further research.

## Author Contributions

D-SY, T-HW, C-YH, Z-GW, Y-HL, and L-FC performed the experiments. H-PY and D-SY performed the statistical analysis. H-PY, N-PW, and L-JL designed the study and drafted the manuscript. All authors participated in writing the manuscript.

## Conflict of Interest Statement

The authors declare that the research was conducted in the absence of any commercial or financial relationships that could be construed as a potential conflict of interest.

## Supplementary Material

FIGURE S1 RNAi silencing analysis of host factors that failed to inhibit ebolavirus transcription- and replication-competent virus-like particle (EBOV-trVLP) replication. Samples (100 μl) of opti-MEM medium containing 1.4 μl siRNA and 4.5 μl HiPerFect were placed in 24-well plates, and a cell suspension (400 μl) containing 1 × 10^5^ 293T cells was added to give a final siRNA concentration of 75 nM. After incubation for 48 h, cells were infected with trVLPs for another 48 h, then total RNA was extracted, and the absolute quantity of EBOV RNA was measured using an EBOV nucleic acid test kit. All qRT-PCR experiments were performed in triplicate and repeated three times independently. Cells not transfected with siRNA but infected with trVLPs served as a blank control, and cells transfected with isotype siRNA and infected with trVLPs served as a negative control.

TABLE S1 TCID50 of trVLPs calculated by Reed–Muench method.

TABLE S2 The gene knock-down efficiency of the most efficient siRNAs chosen for RNAi screening tests.

TABLE S3 Information of each gene chosen for RNA interference screening tests.

## Abbreviations

ChIP: chromatin immunoprecipitation
Co-IP: co-immunoprecipitation
CPE: cytopathic effect
DAPI: 4′,6-diamidino-2-phenylindole
DiI: 1,1′-Dioctadecyl-3,3,3′,3′-Tetramethylindocarbocyanine Perchlorate
DMEM: Dulbecco’s modified Eagle’s medium
EM: electron microscopy
EVD: Ebola virus disease
FBS: fetal calf serum
GP: glycoprotein
HEK: human embryonic kidney
PAGE: polyacrylamide gel electrophoresis
PBS: phosphate-buffered saline
PVDF: polyvinylidene fluoride
qRT-PCR: quantitative real-time reverse transcription polymerase chain reaction
RNAi: RNA interference
RNP: ribonucleoprotein
SDS: sodium dodecyl sulfate
TCID50: 50% tissue culture infective dose
trVLPs: transcription- and replication-competent virus-like particles
VP40: matrix protein 40
Zaire EBOV: Zaire Ebola virus
