# Corrigendum: Chaperones, Membrane Trafficking and Signal Transduction Proteins Regulate Zaire Ebola Virus trVLPs and Interact With trVLP Elements

**DOI:** 10.3389/fmicb.2019.02975

**Published:** 2020-01-14

**Authors:** Dong-Shan Yu, Tian-Hao Weng, Chen-Yu Hu, Zhi-Gang Wu, Yan-Hua Li, Lin-Fang Cheng, Nan-Ping Wu, Lan-Juan Li, Hang-Ping Yao

**Affiliations:** ^1^State Key Laboratory for Diagnosis and Treatment of Infectious Diseases, The First Affiliated Hospital, School of Medicine, Zhejiang University, Hangzhou, China; ^2^Collaborative Innovation Center for Diagnosis and Treatment of Infectious Diseases, The First Affiliated Hospital, School of Medicine, Zhejiang University, Hangzhou, China

**Keywords:** Ebola virus life cycle, trVLPs, RNAi screening, glycoprotein, protein 40, immunoprecipitation

**Figure 3C** on page 7 of this paper erroneously presented two identical western-blot panels (GRP78 and HSPA8).

The corrected Figure 3C presents the **GRP78** and **HSPA8** western-blot panels as originally intended.

**Figure 3:**
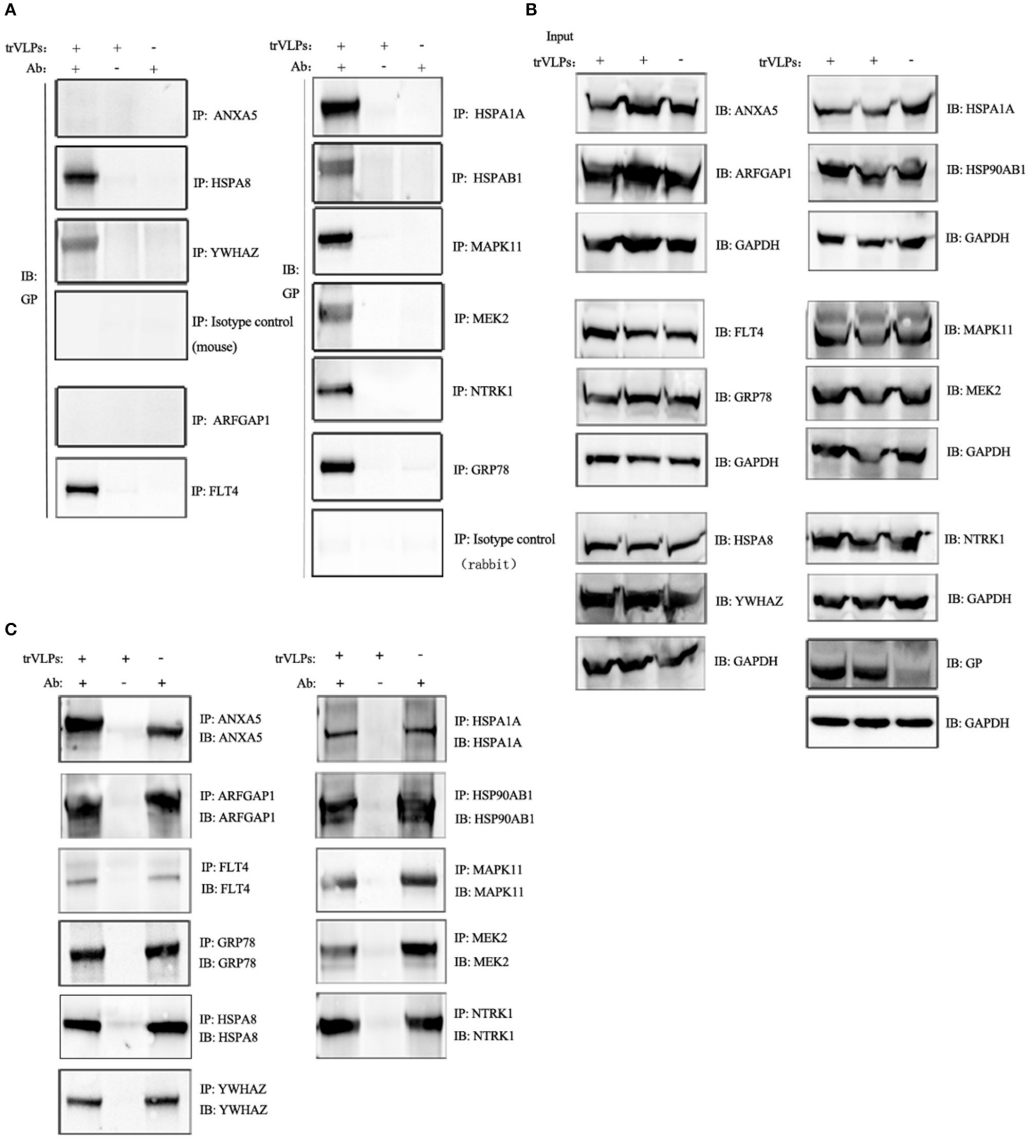
Co-immunoprecipitation (Co-IP) and immunoblot analysis of interaction between target proteins and EBOV-trVLP glycoprotein (GP). **(A)** Co-IP and immunoblot analysis of EBOV trVLP GP. Extracts from HEK 293T cells infected with trVLPs were incubated with antibodies recognizing candidate host proteins plus Protein G beads; pulled-down proteins were detected by western blotting using anti-GP antibody. HSPA1A, MAPK11, NTRK1, GRP78, FLT4, HSPA8, MEK2, HSP90AB1, and YWHAZ interact with GP, whereas ARFGAP1, ANXA5, and isotype controls do not. Extracts incubated with Protein G beads without antibody, and antibodies mixed with Protein G beads and normal cell extracts without trVLPs served as controls. **(B)** Immunoblot analysis of the 11 target proteins before IP and **(C)** after IP. Samples were prepared as described above.

The authors apologize for this error and state that this does not change the scientific conclusions of the article in any way. The original article has been updated.

