# Supplementary figures and images for: Chaperones, Membrane Trafficking and Signal Transduction Proteins Regulate Zaire Ebola Virus trVLPs and Interact With trVLP Elements
Source: Front Microbiol. 2018 Nov 12;9:2724. doi: 10.3389/fmicb.2018.02724 (PMC6240689; doi:10.3389/fmicb.2018.02724)

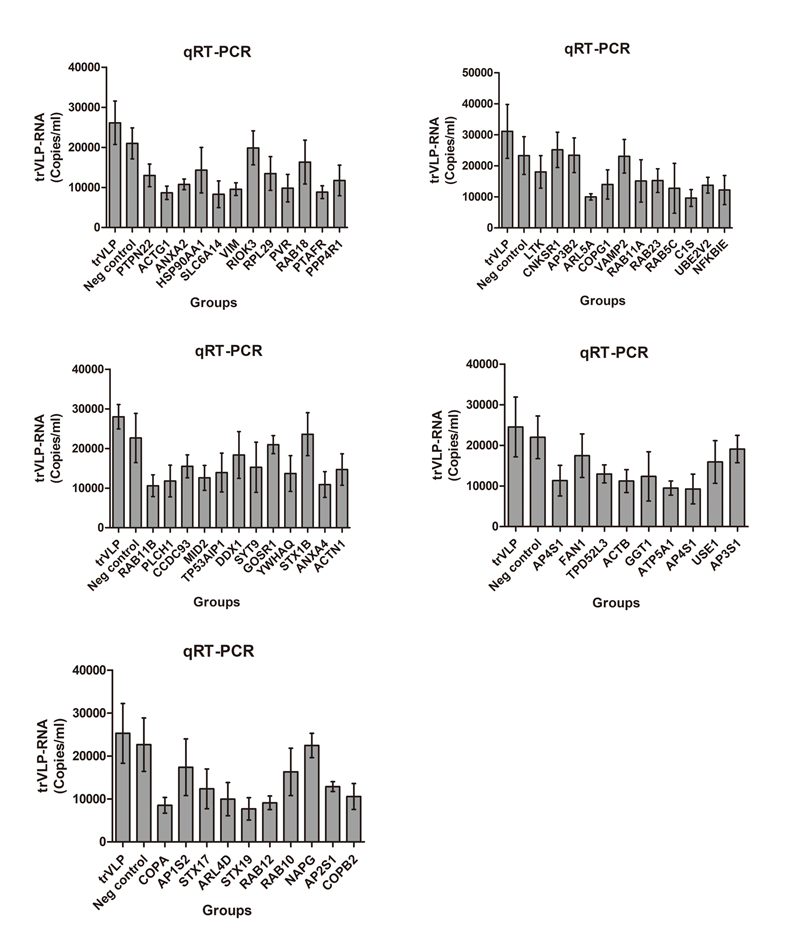

Supplement: FIGURE S1 — RNAi silencing analysis of host factors that failed to inhibit ebolavirus transcription- and replication-competent virus-like particle (EBOV-trVLP) replication. Samples (100 μl) of opti-MEM medium containing 1.4 μl siRNA and 4.5 μl HiPerFect were placed in 24-well plates, and a cell suspension (400 μl) containing 1 × 10^5^ 293T cells was added to give a final siRNA concentration of 75 nM. After incubation for 48 h, cells were infected with trVLPs for another 48 h, then total RNA was extracted, and the absolute quantity of EBOV RNA was measured using an EBOV nucleic acid test kit. All qRT-PCR experiments were performed in triplicate and repeated three times independently. Cells not transfected with siRNA but infected with trVLPs served as a blank control, and cells transfected with isotype siRNA and infected with trVLPs served as a negative control. [file Image_1.TIF]
