# Supplementary material for: Chaperones, Membrane Trafficking and Signal Transduction Proteins Regulate Zaire Ebola Virus trVLPs and Interact With trVLP Elements
Source: Front Microbiol. 2018 Nov 12;9:2724. doi: 10.3389/fmicb.2018.02724 (PMC6240689; doi:10.3389/fmicb.2018.02724)
Supplement: TABLE S1 — TCID50 of trVLPs calculated by Reed–Muench method. [file Table_1.DOCX]

Supplementary table 1. TCID50 of trVLPs calculated by Reed-Muench method.

| Dilution  of trVLPs | Counts of CPE wells | Counts without  CPE wells | Accumulation of CPE wells | Accumulation without CPE wells | Percentage of  CPE wells (%) |
| --- | --- | --- | --- | --- | --- |
| 10^-1^ | 7 | 1 | 19 | 1 | 95.2（20/21） |
| 10^-2^ | 5 | 3 | 12 | 4 | 75.0（12/16） |
| 10^-3^ | 4 | 4 | 7 | 8 | 46.7（7/15） |
| 10^-4^ | 2 | 7 | 3 | 15 | 16.7（3/18） |
| 10^-5^ | 1 | 8 | 1 | 23 | 4.2（1/24） |
| 10^-6^ | 0 | 8 | 0 | 31 | 0（0/31） |
| 10^-7^ | 0 | 8 | 0 |  |  |

Cytopathic effect (CPE) =（75-50）/（75-46.7）= 25 / 28.3 = 0.88

lgTCID50=0.88×（-2）+（-2）= -3.76

TCID50=10^3.76^/ 0.1ml
