# Supplementary material for: Chaperones, Membrane Trafficking and Signal Transduction Proteins Regulate Zaire Ebola Virus trVLPs and Interact With trVLP Elements
Source: Front Microbiol. 2018 Nov 12;9:2724. doi: 10.3389/fmicb.2018.02724 (PMC6240689; doi:10.3389/fmicb.2018.02724)
Supplement: TABLE S2 — The gene knock-down efficiency of the most efficient siRNAs chosen for RNAi screening tests. [file Table_2.DOCX]

Supplementary table 2. The gene knock-down efficiency of the most efficient siRNAs chosen for RNAi screening tests.

| **number** | **siRNAs (product name)** | **Ct（primer：gene）** | **Ct（primer：18s）** | **∆Ct** | **-∆∆Ct** | **2^-∆∆Ct^** |
| --- | --- | --- | --- | --- | --- | --- |
| 1 | negative control | 24.61 | 14.16 | 10.45 |  |  |
|  | Hs_FLT4_9 | 25.16 | 13.01 | 12.15 | -1.7 | 0.307786103 |
| 2 | negative control | 24.50 | 13.72 | 10.78 |  |  |
|  | Hs_RAB11B_4 | 26.97 | 14.54 | 12.43 | -1.65 | 0.318640157 |
| 3 | negative control | 25.83 | 13.84 | 11.99 |  |  |
|  | Hs_PLCH1_2 | 26.27 | 14.28 | 11.99 | 0 | 1.00000 |
| 4 | negative control | 23.13 | 13.65 | 9.48 |  |  |
|  | Hs_CCDC93_6 | 23.53 | 14.00 | 9.53 | -0.05 | 0.965936329 |
| 5 | negative control | 24.76 | 13.76 | 11.00 |  |  |
|  | Hs_MID2_9 | 26.80 | 15.00 | 11.80 | -0.8 | 0.574349177 |
| 6 | negative control | 30.66 | 14.25 | 16.41 |  |  |
|  | Hs_TP53AIP1_1 | 32.63 | 13.08 | 19.55 | -3.14 | 0.113439894 |
| 7 | negative control | 20.62 | 13.62 | 7.00 |  |  |
|  | Hs_DDX1_6 | 23.29 | 15.09 | 8.20 | -1.2 | 0.435275282 |
| 8 | negative control | 25.83 | 14.39 | 11.44 |  |  |
|  | Hs_SYT9_8 | 26.30 | 14.15 | 12.15 | -0.71 | 0.611320139 |
| 9 | negative control | 34.32 | 14.21 | 20.11 |  |  |
|  | Hs_C1S_2 | 36.33 | 13.18 | 23.15 | -3.04 | 0.121581868 |
| 10 | negative control | 19.18 | 14.45 | 4.73 |  |  |
|  | Hs_UBE2V2_8 | 21.08 | 14.27 | 6.81 | -2.08 | 0.236514412 |
| 11 | negative control | 50.09 | 14.14 | 35.95 |  |  |
|  | Hs_PTPN22_3 | 55.00 | 13.26 | 41.74 | -5.79 | 0.018073253 |
| 12 | negative control | 36.00 | 13.92 | 22.08 |  |  |
|  | Hs_SLC6A14_4 | 37.67 | 15.13 | 22.54 | -0.46 | 0.726986259 |
| 13 | negative control | 27.26 | 14.15 | 13.11 |  |  |
|  | Hs_RIOK3_5 | 26.99 | 12.14 | 14.85 | -1.74 | 0.299369676 |
| 14 | negative control | 19.68 | 13.51 | 6.17 |  |  |
|  | Hs_RPL29_10 | 21.74 | 15.61 | 6.13 | 0.04 | 1.028113827 |
| 15 | negative control | 40.36 | 14.16 | 26.20 |  |  |
|  | Hs_PVR_10 | 33.22 | 12.98 | 20.24 | -0.13 | 0.91383145 |
| 16 | negative control | 28.83 | 14.19 | 14.64 |  |  |
|  | Hs_RAB18_8 | 29.55 | 13.35 | 16.20 | -1.56 | 0.339151082 |
| 17 | negative control | 30.08 | 14.15 | 15.93 |  |  |
|  | Hs_PTAFR_1 | 30.95 | 13.71 | 17.24 | -1.31 | 0.40332088 |
| 18 | negative control | 28.87 | 13.67 | 15.20 |  |  |
|  | Hs_PPP4R1_8 | 30.94 | 12.55 | 18.39 | -3.19 | 0.109575715 |
| 19 | negative control | 24.98 | 14.17 | 10.81 |  |  |
|  | Hs_MTMR15_7 | 26.07 | 14.62 | 11.45 | -0.64 | 0.641712949 |
| 20 | negative control | 28.75 | 14.07 | 14.68 |  |  |
|  | Hs_MAPK11_3 | 30.20 | 12.96 | 17.24 | -2.56 | 0.169575541 |
| 21 | negative control | 24.43 | 14.27 | 10.16 |  |  |
|  | Hs_AP4S1_1 | 27.65 | 15.43 | 12.22 | -2.06 | 0.23981603 |
| 22 | negative control | 25.65 | 13.90 | 11.75 |  |  |
|  | Hs_MEK2_6 | 27.38 | 13.02 | 14.36 | -2.61 | 0.163799175 |
| 23 | negative control | 28.59 | 13.91 | 14.68 |  |  |
|  | Hs_NFKBIE_1 | 29.31 | 12.81 | 16.50 | -1.82 | 0.283220971 |
| 24 | negative control | 30.13 | 13.77 | 16.36 |  |  |
|  | Hs_LTK_7 | 31.92 | 13.62 | 18.30 | -1.94 | 0.26061644 |
| 25 | negative control | 38.35 | 13.83 | 24.52 |  |  |
|  | Hs_NYD-SP25_10 | 41.18 | 12.96 | 28.22 | -3.7 | 0.076946526 |
| 26 | negative control | 28.74 | 14.42 | 14.32 |  |  |
|  | Hs_CNKSR1_5 | 29.29 | 15.61 | 13.68 | 0.64 | 1.558329159 |
| 27 | negative control | 19.87 | 13.78 | 6.09 |  |  |
|  | Hs_COPG_1 | 21.36 | 13.19 | 8.17 | -2.08 | 0.236514412 |
| 28 | negative control | 26.96 | 14.27 | 12.69 |  |  |
|  | Hs_MDS032_3 | 28.13 | 14.21 | 13.92 | -1.23 | 0.426317446 |
| 29 | negative control | 21.18 | 14.19 | 6.99 |  |  |
|  | Hs_RAB11A_6 | 22.72 | 13.76 | 8.96 | -1.97 | 0.255253031 |
| 30 | negative control | 21.68 | 14.58 | 7.10 |  |  |
|  | Hs_AP3S1_9 | 24.16 | 14.35 | 9.81 | -2.71 | 0.152830035 |
| 31 | negative control | 21.86 | 14.11 | 7.75 |  |  |
|  | Hs_ARL5_4 | 24.71 | 13.90 | 10.81 | -3.06 | 0.119908015 |
| 32 | negative control | 21.66 | 15.66 | 6.00 |  |  |
|  | Hs_VAMP2_5 | 23.48 | 13.73 | 9.75 | -3.75 | 0.074325445 |
| 33 | negative control | 33.34 | 15.64 | 17.70 |  |  |
|  | Hs_RAB23_1 | 34.80 | 13.98 | 20.82 | -3.12 | 0.115023456 |
| 34 | negative control | 24.43 | 14.23 | 10.20 |  |  |
|  | Hs_GOSR1_2 | 25.96 | 14.98 | 10.98 | -0.78 | 0.582366793 |
| 35 | negative control | 30.54 | 15.68 | 14.86 |  |  |
|  | Hs_STX1B2_1 | 34.15 | 14.14 | 20.01 | -5.15 | 0.028164077 |
| 36 | negative control | 20.16 | 14.52 | 5.64 |  |  |
|  | Hs_AP2S1_4 | 22.29 | 14.67 | 7.62 | -1.98 | 0.25348987 |
| 37 | negative control | 26.46 | 14.57 | 11.89 |  |  |
|  | Hs_AP3B2_8 | 27.97 | 16.34 | 11.63 | 0.26 | 1.197478705 |
| 38 | negative control | 30.25 | 13.34 | 16.91 |  |  |
|  | Hs_COPA_9 | 32.77 | 14.45 | 18.32 | -1.41 | 0.376311687 |
| 39 | negative control | 20.77 | 15.77 | 5.00 |  |  |
|  | Hs_AP1S2_7 | 21.79 | 14.16 | 7.63 | -2.63 | 0.161544104 |
| 40 | negative control | 25.17 | 15.70 | 9.47 |  |  |
|  | Hs_STX17_3 | 27.71 | 14.44 | 13.27 | -3.8 | 0.071793647 |
| 41 | negative control | 25.47 | 14.32 | 11.15 |  |  |
|  | Hs_ARL4D_2 | 26.21 | 14.23 | 11.98 | -0.83 | 0.562529242 |
| 42 | negative control | 30.03 | 24.65 | 5.38 |  |  |
|  | Hs_MGC21382_1 | 31.03 | 27.03 | 4.00 | 1.38 | 2.602683711 |
| 43 | negative control | 22.74 | 14.29 | 8.45 |  |  |
|  | Hs_RAB12_5 | 24.73 | 14.81 | 9.92 | -1.47 | 0.360982299 |
| 44 | negative control | 21.52 | 14.40 | 7.12 |  |  |
|  | Hs_RAB10_5 | 22.50 | 15.07 | 7.43 | -0.31 | 0.806641759 |
| 45 | negative control | 24.83 | 15.95 | 8.88 |  |  |
|  | Hs_NAPG_7 | 26.45 | 15.24 | 11.21 | -2.33 | 0.198884121 |
| 46 | negative control | 27.86 | 15.81 | 12.05 |  |  |
|  | Hs_HSPA8_10 | 28.85 | 14.89 | 13.96 | -1.91 | 0.266092546 |
| 47 | negative control | 21.60 | 13.27 | 8.33 |  |  |
|  | Hs_HSP90AB1_5 | 22.80 | 14.18 | 8.62 | -0.29 | 0.817902059 |
| 48 | negative control | 25.12 | 13.70 | 11.42 |  |  |
|  | Hs_ANXA2_10 | 25.73 | 14.22 | 11.51 | -0.09 | 0.939522749 |
| 49 | negative control | 30.59 | 13.80 | 16.79 |  |  |
|  | Hs_HSP90AA1_2 | 30.41 | 14.21 | 16.20 | 0.59 | 1.505246747 |
| 50 | negative control | 20.53 | 14.15 | 6.38 |  |  |
|  | Hs_ARFGAP1_8 | 20.70 | 14.73 | 5.97 | 0.41 | 1.328685814 |
| 51 | negative control | 19.45 | 13.71 | 5.74 |  |  |
|  | Hs_VIM_11 | 36.67 | 14.39 | 22.28 | -16.54 | 0.000010495 |
| 52 | negative control | 31.22 | 13.79 | 17.43 |  |  |
|  | Hs_NTRK1_1 | 31.39 | 14.69 | 16.70 | 0.73 | 1.658639092 |
| 53 | negative control | 16.29 | 14.16 | 2.13 |  |  |
|  | Hs_ACTG1_2 | 17.55 | 13.95 | 3.60 | -1.47 | 0.360982299 |
| 54 | negative control | 36.40 | 13.67 | 22.73 |  |  |
|  | Hs_HSPA1A_9 | 37.42 | 14.78 | 22.64 | 0.09 | 1.064370182 |
| 55 | negative control | 21.42 | 13.83 | 7.59 |  |  |
|  | Hs_ANXA5_6 | 23.33 | 14.96 | 8.37 | -0.78 | 0.582366793 |
| 56 | negative control | 16.51 | 13.81 | 2.70 |  |  |
|  | Hs_ACTB_9 | 18.08 | 15.02 | 3.06 | -0.36 | 0.77916458 |
| 57 | negative control | 25.34 | 13.88 | 11.46 |  |  |
|  | Hs_GGT1_10 | 26.34 | 15.04 | 11.30 | 0.16 | 1.117287138 |
| 58 | negative control | 19.43 | 13.79 | 5.64 |  |  |
|  | Hs_ATP5A1_5 | 21.04 | 14.95 | 6.09 | -0.45 | 0.732042848 |
| 59 | negative control | 18.92 | 13.65 | 5.27 |  |  |
|  | Hs_YWHAZ_3 | 21.15 | 13.50 | 7.65 | -2.38 | 0.192109398 |
| 60 | negative control | 35.79 | 13.79 | 22.00 |  |  |
|  | Hs_ANXA4_6 | 35.72 | 13.62 | 22.10 | -0.1 | 0.933032992 |
| 61 | negative control | 29.00 | 13.74 | 15.26 |  |  |
|  | Hs_HSPA5_7 | 20.59 | 13.74 | 6.85 | -1.58 | 0.334481889 |
| 62 | negative control | 19.29 | 13.84 | 5.45 |  |  |
|  | Hs_YWHAQ_6 | 22.04 | 13.98 | 8.06 | -2.61 | 0.163799175 |
| 63 | negative control | 22.28 | 13.78 | 8.50 |  |  |
|  | Hs_ACTN1_10 | 28.87 | 16.30 | 12.57 | -4.07 | 0.059539875 |
| 64 | negative control | 19.90 | 14.42 | 5.48 |  |  |
|  | Hs_COPB2_6 | 21.10 | 16.36 | 4.74 | 0.74 | 1.670175839 |
| 65 | negative control | 21.44 | 14.33 | 7.11 |  |  |
|  | Hs_RAB5C_5 | 23.10 | 14.62 | 8.48 | -1.37 | 0.386891248 |

18s, 18sRNA, was treated as control.
