# Supplementary material for: Chaperones, Membrane Trafficking and Signal Transduction Proteins Regulate Zaire Ebola Virus trVLPs and Interact With trVLP Elements
Source: Front Microbiol. 2018 Nov 12;9:2724. doi: 10.3389/fmicb.2018.02724 (PMC6240689; doi:10.3389/fmicb.2018.02724)
Supplement: TABLE S3 — Information of each gene chosen for RNA interference screening tests. [file Table_3.DOCX]

Supplementary table 3. Information of each gene chosen for RNA interference screening tests.

| **Entrez Gene Id** | **NCBI gene symbol** | **Gene Description（references）** | **mRNA Accessions** | **siRNA Target Sequence** | **Product Id** | **Product Name** |
| --- | --- | --- | --- | --- | --- | --- |
| 2324 | FLT4 | fms-related tyrosine kinase 4(Clauss, 2000; Simpson et al., 2012) | NM_002020 NM_182925 | CACGCTCTTGGTCAACAGGAA | Qiagen,  SI02225454 | Hs_FLT4_9 |
| 9230 | RAB11B | RAB11B, member RAS oncogene family(Grimsey et al., 2016) | NM_004218 | CGAGTTCAACCTGGAGAGCAA | Qiagen, SI00061166 | Hs_RAB11B_4 |
| 23007 | PLCH1 | phospholipase C, eta 1(Stewart et al., 2005) | NM_001130960 NM_001130961 NM_014996 | CACGGTCTAAATCTTACAGTA | Qiagen, SI00686217 | Hs_PLCL1_2 |
| 54520 | CCDC93 | coiled-coil domain containing 93(Loaiza et al., 2017) | NM_019044 | CAGCATCTAGCTCAAGGTAGA | Qiagen, SI05122929 | Hs_CCDC93_6 |
| 11043 | MID2 | midline 2(Boding et al., 2015) | NM_012216 NM_052817 | TACACCTAAGTTAGCGTTCAA | Qiagen, SI05096133 | Hs_MID2_9 |
| 63970 | TP53AIP1 | tumor protein p53 regulated apoptosis inducing protein 1(Matsuda et al., 2002) | NM_001195194 NM_001195195 NM_001251964 NM_022112 | AACAGAAAGCACCGTCGAGAA | Qiagen, SI05461743 | Hs_TP53AIP1_1 |
| 1653 | DDX1 | DEAD box helicase 1(Lin et al., 2014) | NM_004939 | CACGGTGTTCCTTATGTTATA | Qiagen, SI02634107 | Hs_DDX1_6 |
| 143425 | SYT9 | synaptotagmin IX(Zhang et al., 2010) | NM_175733 | CCCGGCATAATTCAATCCGAA | Qiagen, SI04302578 | Hs_SYT9_8 |
| 716 | C1S | complement component 1, s subcomponent(Brier et al., 2010) | NM_001734 NM_201442 | AGGATCCGATGCAGATATTAA | Qiagen, SI00027363 | Hs_C1S_2 |
| 7336 | UBE2V2 | ubiquitin-conjugating enzyme E2 variant 2(Chai et al., 2015) | NM_003350 | AACAACCTTCTACTCATGTTA | Qiagen, SI05096595 | Hs_UBE2V2_8 |
| 26191 | PTPN22 | protein tyrosine phosphatase, non-receptor type 22 (Perri et al., 2017; Spalinger et al., 2017) | NM_001193431 NM_012411 NM_015967 | TACGTAATGCCTCTAATGTAA | Qiagen, SI00112777 | Hs_PTPN22_3 |
| 11254 | SLC6A14 | solute carrier family 6 (amino acid transporter), member 14(Coothankandaswamy et al., 2016) | NM_007231 | AGGCGCCTTCTTGATACCTTA | Qiagen, SI00095739 | Hs_SLC6A14_4 |
| 8780 | RIOK3 | RIO kinase 3(Takashima et al., 2015; Zhang et al., 2018) | NM_003831 NM_145906 | ATGCGGCAGTTATATCATGAA | Qiagen, SI02223396 | Hs_RIOK3_5 |
| 6159 | RPL29 | ribosomal protein L29(Jones et al., 2013) | NM_000992 | CAACATGAGGACAGAAGGACT | Qiagen, SI05124882 | Hs_RPL29_10 |
| 5817 | PVR | poliovirus receptor(Strauss et al., 2015) | NM_001135768 NM_001135769 NM_001135770 NM_006505 | TAGGCTCACAGTTACAGTTTA | Qiagen, SI04992442 | Hs_PVR_10 |
| 22931 | RAB18 | RAB18, member RAS oncogene family(Dansako et al., 2014) | NM_001256410 NM_001256411 NM_001256412 NM_001256415 NM_021252 | CCAGGCCAATTTATAACTAAA | Qiagen, SI02662709 | Hs_RAB18_8 |
| 5724 | PTAFR | platelet-activating factor receptor(Ji et al., 2016) | NM_000952 NM_001164721 NM_001164722 NM_001164723 | CGCCTGTACCCTTGCAAGAAA | Qiagen, SI00019075 | Hs_PTAFR_1 |
| 9989 | PPP4R1 | protein phosphatase 4, regulatory subunit 1(Abdul-Sada et al., 2017) | NM_001042388 NM_005134 | TCAGGCGTTGTTAGATCAGTA | Qiagen, SI03113026 | Hs_PPP4R1_8 |
| 22909 | FAN1 | FANCD2/FANCI-associated nuclease 1(Jin and Cho, 2017) | NM_001146094 NM_001146095 NM_001146096 NM_014967 | TTGGGAAGCCTAGCATCTAAA | Qiagen, SI04159246 | Hs_MTMR15_7 |
| 5600 | MAPK11 | mitogen-activated protein kinase 11(Beenstock et al., 2014; Hayakawa et al., 2017) | NM_002751 NM_138993 | CTGAGCGACGAGCACGTTCAA | Qiagen, SI00606060 | Hs_MAPK11_3 |
| 11154 | AP4S1 | adaptor-related protein complex 4, sigma 1 subunit(Tuysuz et al., 2014) | NM_001128126 NM_001254726 NM_001254727 NM_001254728 NM_001254729 NM_007077 | ATGCTCTTTCATTGAATATAA | Qiagen, SI00297703 | Hs_AP4S1_1 |
| 5605 | MEK2 | Dual specificity mitogen-activated protein kinase kinase 2(Aoidi et al., 2016) | NM_030662 | TTACGAGGATGTTAAACTTAA | Qiagen, SI00605647 | Hs_MEK2_6 |
| 4794 | NFKBIE | nuclear factor of kappa light polypeptide gene enhancer in B-cells inhibitor, epsilon(Kohda et al., 2016) | NM_004556 | CTGGCTGTACATCTGGACCAA | Qiagen, SI00065142 | Hs_NFKBIE_1 |
| 4058 | LTK | leukocyte receptor tyrosine kinase(Reshetnyak et al., 2015) | NM_001135685 NM_002344 NM_206961 | CACTGGATTGCTCTCCCATTA | Qiagen, SI03649457 | Hs_LTK_7 |
| 89882 | TPD52L3 | tumor protein D52-like 3(Cao et al., 2006) | NM_001001874 NM_001001875 NM_033516 | CACGTTAAATCAAGGAAGGAA | Qiagen, SI00604709 | Hs_NYD-SP25_10 |
| 10256 | CNKSR1 | connector enhancer of kinase suppressor of Ras 1(Fischer et al., 2015) | NM_006314 NR_023345 | ACCCATGACTTCCAGAGCATA | Qiagen, SI02665411 | Hs_CNKSR1_5 |
| 22820 | COPG1 | coatomer protein complex, subunit gamma 1(Pan et al., 2013) | NM_016128 | CCGAGCCACCTTCTACCTAAA | Qiagen, SI00113666 | Hs_COPG_1 |
| 55850 | USE1 | unconventional SNARE in the ER 1 homolog (S. cerevisiae)(Aichem et al., 2010) | NM_018467 | CCCGGAGCCTCAAGACCAATA | Qiagen, SI04363674 | Hs_MDS032_3 |
| 8766 | RAB11A | RAB11A, member RAS oncogene family(Hook et al., 2014) | NM_001206836 NM_004663 | CGAAATGAGTTTAATCTGGAA | Qiagen, SI02655247 | Hs_RAB11A_6 |
| 1176 | AP3S1 | adaptor-related protein complex 3, sigma 1 subunit(Petrenko et al., 2006) | NM_001002924 NM_001284 | AAGGTTCACAATATTCTTGCA | Qiagen, SI03036621 | Hs_AP3S1_9 |
| 26225 | ARL5A | ADP-ribosylation factor-like 5A(Rosa-Ferreira et al., 2015) | NM_001037174 NM_012097 NM_177985 | CAAGTTAATGGCATTGATTTA | Qiagen, SI00303562 | Hs_ARL5_4 |
| 6844 | VAMP2 | vesicle-associated membrane protein 2 (synaptobrevin 2)(Caceres et al., 2014) | NM_014232 | AACAAGCGCAGCCAAGCTCAA | Qiagen, SI03027241 | Hs_VAMP2_5 |
| 51715 | RAB23 | RAB23, member RAS oncogene family(Zheng et al., 2017) | NM_016277 NM_183227 | GAGCGACAAATTCAAGTTAAT | Qiagen, SI03103030 | Hs_RAB23_9 |
| 9527 | GOSR1 | golgi SNAP receptor complex member 1(Xu et al., 2002) | NM_001007024 NM_001007025 NM_004871 | CATGATAGTGTTGAAGCCTAA | Qiagen, SI00020622 | Hs_GOSR1_2 |
| 112755 | STX1B | syntaxin 1B(Mishima et al., 2014) | NM_052874 | AAGGTTCGGTCCAAATTGAAA | Qiagen, SI00147000 | Hs_STX1B2_1 |
| 1175 | AP2S1 | adaptor-related protein complex 2, sigma 1 subunit(Rogers et al., 2014) | NM_004069 NM_021575 | CCGAGACGCCAAACACACCAA | Qiagen, SI00297472 | Hs_AP2S1_4 |
| 8120 | AP3B2 | adaptor-related protein complex 3, beta 2 subunit(Evstratova et al., 2014) | NM_004644 | GCGCATCGACCTGATTCACAA | Qiagen, SI04262272 | Hs_AP3B2_8 |
| 1314 | COPA | coatomer protein complex, subunit alpha(Watkin et al., 2015) | NM_001098398 NM_004371 | TACCCTCTGTGTACTCAGAAA | Qiagen, SI05016375 | Hs_COPA_9 |
| 8905 | AP1S2 | adaptor-related protein complex 1, sigma 2 subunit(Candiello et al., 2016) | NM_003916 | ATGGCACATCATATACATGTA | Qiagen, SI02632245 | Hs_AP1S2_7 |
| 55014 | STX17 | syntaxin 17(Hegedus et al., 2013; Kumar et al., 2018) | NM_017919 | ACCCTGAATTGGAGACCTTAA | Qiagen, SI00120155 | Hs_STX17_3 |
| 379 | ARL4D | ADP-ribosylation factor-like 4D(Li et al., 2012) | NM_001661 | CAGGGCCTTGAGCGCCTCTAT | Qiagen, SI04328996 | Hs_ARL4D_4 |
| 415117 | STX19 | syntaxin 19(Wang et al., 2006) | NM_001001850 | GAGAAGGTTTAGTCTACTTAA | Qiagen, SI02624867 | Hs_MGC21382_7 |
| 201475 | RAB12 | RAB12, member RAS oncogene family(Efergan et al., 2016) | NM_001025300 XM_113967 | CAGCATTACCTCAGCTTATTA | Qiagen, SI00486206 | Hs_RAB12_5 |
| 10890 | RAB10 | RAB10, member RAS oncogene family(Liu and Grant, 2015) | NM_016131 | AAGGGACAAACTAGTAGGTTT | Qiagen, SI00113708 | Hs_RAB10_3 |
| 8774 | NAPG | N-ethylmaleimide-sensitive factor attachment protein, gamma(Brondijk et al., 2002) | NM_003826 | CAGCAGCGCAGCTATGCTTTA | Qiagen, SI05051403 | Hs_NAPG_8 |
| 3312 | HSPA8 | heat shock 70kDa protein 8(Dong et al., 2018) | NM_006597 NM_153201 | AAGGACCTAAATTCGTAGCAA | Qiagen, SI02661477 | Hs_HSPA8_6 |
| 3326 | HSP90AB1 | heat shock protein 90kDa alpha (cytosolic), class B member 1(Gao et al., 2014; Srisutthisamphan et al., 2018) | NM_007355 NM_001271969 NM_001271970 NM_001271971 NM_001271972 | CAAGAATGATAAGGCAGTTAA | Qiagen, SI02780561 | Hs_HSP90AB1_5 |
| 302 | ANXA2 | annexin A2(Grindheim et al., 2017) | NM_001002857 NM_001002858 NM_001136015 NM_004039 | CACGGCCTGAGCGTCCAGAAA | Qiagen, SI03060855 | Hs_ANXA2_10 |
| 3320 | HSP90AA1 | heat shock protein 90kDa alpha (cytosolic), class A member 1(Katoh et al., 2017) | NM_001017963 NM_005348 | TGCACTGTAAGACGTATGTAA | Qiagen, SI03117814 | Hs_HSP90AA1_2 |
| 375 | ARFGAP1 | ADP-ribosylation factor GTPase-activating protein 1(Weimer et al., 2008) | NM_001024226 NM_001024227 NM_001024228 NM_001658 | CACCATAGGCTTCAACGTGGA | Qiagen, SI02654470 | Hs_ARF1_8 |
| 7431 | VIM | vimentin(Schafer et al., 2017) | NM_003380 | AAGATCCTGCTGGCCGAGCTC | Qiagen, SI02655198 | Hs_VIM_11 |
| 4914 | NTRK1 | neurotrophic tyrosine kinase, receptor, type 1(Anwar et al., 2011) | NM_001007204 NM_001007792 NM_001012331 NM_002529 | CGAGAGCATCCTGTACCGTAA | Qiagen, SI00038983 | Hs_NTRK1_1 |
| 71 | ACTG1 | actin, gamma 1(Shum et al., 2011) | NM_001199954 NM_001614 NR_037688 | ATGGGTTAATTGAGAATAGAA | Qiagen, SI02652083 | Hs_ACTG1_2 |
| 3303 | HSPA1A | heat shock 70kDa protein 1A(Deffit and Blum, 2015) | NM_005345 | TCCGGTTTCTACATGCAGAGA | Qiagen, SI04364136 | Hs_HSPA1A_9 |
| 308 | ANXA5 | annexin A5(Jheng et al., 2016; de Jong et al., 2018) | NM_001154 | CGCGAGACTTCTGGCAATTTA | SI04159687 | Hs_ANXA5_6 |
| 60 | ACTB | actin, beta(Wu et al., 2016) | NM_001101 | GCCGAGGACTTTGATTGCACA | Qiagen, SI04287759 | Hs_ACTB_9 |
| 2678 | GGT1 | gamma-glutamyltransferase 1(Jinnouchi et al., 2015) | NM_001032364 NM_001032365 NM_005265 NM_013421 NM_013430 | CCAGGAGGACAAGGCTGACAA | Qiagen, SI04146009 | Hs_GGT1_10 |
| 498 | ATP5A1 | ATP synthase, H+ transporting, mitochondrial F1 complex, alpha subunit 1, cardiac muscle(Bernardi et al., 2015) | NM_001001935 NM_001001937 NM_004046 | TTGGCTGGATTTGAAGCTTAA | Qiagen, SI02776991 | Hs_ATP5A1_5 |
| 7534 | YWHAZ | tyrosine 3-monooxygenase/tryptophan 5-monooxygenase activation protein, zeta(Tong et al., 2016) | NM_001135699 NM_001135700 NM_001135701 NM_001135702 NM_003406 NM_145690 | CAGGTTTATGTTACTTCTATT | Qiagen, SI00764813 | Hs_YWHAZ_3 |
| 307 | ANXA4 | annexin A4(Heinick et al., 2015) | NM_001153 | AAGGATATCACAGAAGGATAT | Qiagen, SI04149481 | Hs_ANXA4_6 |
| 3309 | HSPA5 | heat shock 70kDa protein 5 (glucose-regulated protein, 78kDa)(Jheng et al., 2016) | NM_005347 | TGGGATAAGGAAACACTTCTA | Qiagen, SI02781016 | Hs_HSPA5_7 |
| 10971 | YWHAQ | tyrosine 3-monooxygenase/tryptophan 5-monooxygenase activation protein, theta | NM_006826 | CAAACGATAGATAATTCCCAA | Qiagen, SI00092197 | Hs_YWHAQ_6 |
| 87 | ACTN1 | actinin, alpha 1(Ben-Addi et al., 2014) | NM_001102 NM_001130004 NM_001130005 | CCGGCCCGAGCTGATTGACTA | Qiagen, SI02654414 | Hs_ACTN1_8 |
| 9276 | COPB2 | coatomer protein complex, subunit beta 2 (beta prime)(Mi et al., 2016) | NM_004766 NR_023350 | AAACAGATCATTATTATATAT | Qiagen, SI05108747 | Hs_COPB2_6 |
| 5878 | RAB5C | RAB5C, member RAS oncogene family (Radhakrishnan et al., 2012; Simpson et al., 2012; Hook et al., 2014) | NM_001252039 NM_004583 NM_201434 | CACCATGATTTCTCCATATAA | Qiagen, SI02663073 | Hs_RAB5C_5 |

**References：**

Abdul-Sada, H., Muller, M., Mehta, R., Toth, R., Arthur, J.S.C., Whitehouse, A., et al. (2017). The PP4R1 sub-unit of protein phosphatase PP4 is essential for inhibition of NF-kappaB by merkel polyomavirus small tumour antigen. *Oncotarget* 8(15)**,** 25418-25432. doi: 10.18632/oncotarget.15836.

Aichem, A., Pelzer, C., Lukasiak, S., Kalveram, B., Sheppard, P.W., Rani, N., et al. (2010). USE1 is a bispecific conjugating enzyme for ubiquitin and FAT10, which FAT10ylates itself in cis. *Nat Commun* 1**,** 13. doi: 10.1038/ncomms1012.

Anwar, A., Hosoya, T., Leong, K.M., Onogi, H., Okuno, Y., Hiramatsu, T., et al. (2011). The kinase inhibitor SFV785 dislocates dengue virus envelope protein from the replication complex and blocks virus assembly. *PLoS One* 6(8)**,** e23246. doi: 10.1371/journal.pone.0023246.

Aoidi, R., Maltais, A., and Charron, J. (2016). Functional redundancy of the kinases MEK1 and MEK2: Rescue of the Mek1 mutant phenotype by Mek2 knock-in reveals a protein threshold effect. *Sci Signal* 9(412)**,** ra9. doi: 10.1126/scisignal.aad5658.

Beenstock, J., Ben-Yehuda, S., Melamed, D., Admon, A., Livnah, O., Ahn, N.G., et al. (2014). The p38beta mitogen-activated protein kinase possesses an intrinsic autophosphorylation activity, generated by a short region composed of the alpha-G helix and MAPK insert. *J Biol Chem* 289(34)**,** 23546-23556. doi: 10.1074/jbc.M114.578237.

Ben-Addi, A., Mambole-Dema, A., Brender, C., Martin, S.R., Janzen, J., Kjaer, S., et al. (2014). IkappaB kinase-induced interaction of TPL-2 kinase with 14-3-3 is essential for Toll-like receptor activation of ERK-1 and -2 MAP kinases. *Proc Natl Acad Sci U S A* 111(23)**,** E2394-2403. doi: 10.1073/pnas.1320440111.

Bernardi, P., Di Lisa, F., Fogolari, F., and Lippe, G. (2015). From ATP to PTP and Back: A Dual Function for the Mitochondrial ATP Synthase. *Circ Res* 116(11)**,** 1850-1862. doi: 10.1161/circresaha.115.306557.

Boding, L., Hansen, A.K., Meroni, G., Levring, T.B., Woetmann, A., Odum, N., et al. (2015). MID2 can substitute for MID1 and control exocytosis of lytic granules in cytotoxic T cells. *Apmis* 123(8)**,** 682-687. doi: 10.1111/apm.12402.

Brier, S., Pflieger, D., Le Mignon, M., Bally, I., Gaboriaud, C., Arlaud, G.J., et al. (2010). Mapping surface accessibility of the C1r/C1s tetramer by chemical modification and mass spectrometry provides new insights into assembly of the human C1 complex. *J Biol Chem* 285(42)**,** 32251-32263. doi: 10.1074/jbc.M110.149112.

Brondijk, T.H., Fiegen, D., Richardson, D.J., and Cole, J.A. (2002). Roles of NapF, NapG and NapH, subunits of the Escherichia coli periplasmic nitrate reductase, in ubiquinol oxidation. *Mol Microbiol* 44(1)**,** 245-255.

Caceres, P.S., Mendez, M., and Ortiz, P.A. (2014). Vesicle-associated membrane protein 2 (VAMP2) but Not VAMP3 mediates cAMP-stimulated trafficking of the renal Na+-K+-2Cl- co-transporter NKCC2 in thick ascending limbs. *J Biol Chem* 289(34)**,** 23951-23962. doi: 10.1074/jbc.M114.589333.

Candiello, E., Kratzke, M., Wenzel, D., Cassel, D., and Schu, P. (2016). AP-1/sigma1A and AP-1/sigma1B adaptor-proteins differentially regulate neuronal early endosome maturation via the Rab5/Vps34-pathway. *Sci Rep* 6**,** 29950. doi: 10.1038/srep29950.

Cao, Q., Chen, J., Zhu, L., Liu, Y., Zhou, Z., Sha, J., et al. (2006). A testis-specific and testis developmentally regulated tumor protein D52 (TPD52)-like protein TPD52L3/hD55 interacts with TPD52 family proteins. *Biochem Biophys Res Commun* 344(3)**,** 798-806. doi: 10.1016/j.bbrc.2006.03.208.

Chai, F., Li, H.Y., Wang, W., Zhu, X.J., Li, Y., Wang, S., et al. (2015). Subcellular quantitative proteomic analysis reveals host proteins involved in human cytomegalovirus infection. *Biochim Biophys Acta* 1854(8)**,** 967-978. doi: 10.1016/j.bbapap.2015.04.016.

Clauss, M. (2000). Molecular biology of the VEGF and the VEGF receptor family. *Semin Thromb Hemost* 26(5)**,** 561-569. doi: 10.1055/s-2000-13213.

Coothankandaswamy, V., Cao, S., Xu, Y., Prasad, P.D., Singh, P.K., Reynolds, C.P., et al. (2016). Amino acid transporter SLC6A14 is a novel and effective drug target for pancreatic cancer. *Br J Pharmacol* 173(23)**,** 3292-3306. doi: 10.1111/bph.13616.

Dansako, H., Hiramoto, H., Ikeda, M., Wakita, T., and Kato, N. (2014). Rab18 is required for viral assembly of hepatitis C virus through trafficking of the core protein to lipid droplets. *Virology* 462-463**,** 166-174. doi: 10.1016/j.virol.2014.05.017.

de Jong, R.C.M., Pluijmert, N.J., de Vries, M.R., Pettersson, K., Atsma, D.E., Jukema, J.W., et al. (2018). Annexin A5 reduces infarct size and improves cardiac function after myocardial ischemia-reperfusion injury by suppression of the cardiac inflammatory response. *Sci Rep* 8(1)**,** 6753. doi: 10.1038/s41598-018-25143-y.

Deffit, S.N., and Blum, J.S. (2015). A central role for HSC70 in regulating antigen trafficking and MHC class II presentation. *Mol Immunol* 68(2 Pt A)**,** 85-88. doi: 10.1016/j.molimm.2015.04.007.

Dong, Q., Men, R., Dan, X., Chen, Y., Li, H., Chen, G., et al. (2018). Hsc70 regulates the IRES activity and serves as an antiviral target of enterovirus A71 infection. *Antiviral Res* 150**,** 39-46. doi: 10.1016/j.antiviral.2017.11.020.

Efergan, A., Azouz, N.P., Klein, O., Noguchi, K., Rothenberg, M.E., Fukuda, M., et al. (2016). Rab12 Regulates Retrograde Transport of Mast Cell Secretory Granules by Interacting with the RILP-Dynein Complex. *J Immunol* 196(3)**,** 1091-1101. doi: 10.4049/jimmunol.1500731.

Evstratova, A., Chamberland, S., Faundez, V., and Toth, K. (2014). Vesicles derived via AP-3-dependent recycling contribute to asynchronous release and influence information transfer. *Nat Commun* 5**,** 5530. doi: 10.1038/ncomms6530.

Fischer, A., Brummer, T., Warscheid, B., and Radziwill, G. (2015). Differential tyrosine phosphorylation controls the function of CNK1 as a molecular switch in signal transduction. *Biochim Biophys Acta* 1853(11 Pt A)**,** 2847-2855. doi: 10.1016/j.bbamcr.2015.08.014.

Gao, J., Xiao, S., Liu, X., Wang, L., Zhang, X., Ji, Q., et al. (2014). Inhibition of HSP90 attenuates porcine reproductive and respiratory syndrome virus production in vitro. *Virol J* 11**,** 17. doi: 10.1186/1743-422x-11-17.

Grimsey, N.J., Coronel, L.J., Cordova, I.C., and Trejo, J. (2016). Recycling and Endosomal Sorting of Protease-activated Receptor-1 Is Distinctly Regulated by Rab11A and Rab11B Proteins. *J Biol Chem* 291(5)**,** 2223-2236. doi: 10.1074/jbc.M115.702993.

Grindheim, A.K., Saraste, J., and Vedeler, A. (2017). Protein phosphorylation and its role in the regulation of Annexin A2 function. *Biochim Biophys Acta* 1861(11 Pt A)**,** 2515-2529. doi: 10.1016/j.bbagen.2017.08.024.

Hayakawa, M., Hayakawa, H., Petrova, T., Ritprajak, P., Sutavani, R.V., Jimenez-Andrade, G.Y., et al. (2017). Loss of Functionally Redundant p38 Isoforms in T Cells Enhances Regulatory T Cell Induction. *J Biol Chem* 292(5)**,** 1762-1772. doi: 10.1074/jbc.M116.764548.

Hegedus, K., Takats, S., Kovacs, A.L., and Juhasz, G. (2013). Evolutionarily conserved role and physiological relevance of a STX17/Syx17 (syntaxin 17)-containing SNARE complex in autophagosome fusion with endosomes and lysosomes. *Autophagy* 9(10)**,** 1642-1646. doi: 10.4161/auto.25684.

Heinick, A., Husser, X., Himmler, K., Kirchhefer, U., Nunes, F., Schulte, J.S., et al. (2015). Annexin A4 is a novel direct regulator of adenylyl cyclase type 5. *Faseb j* 29(9)**,** 3773-3787. doi: 10.1096/fj.14-269837.

Hook, L.M., Grey, F., Grabski, R., Tirabassi, R., Doyle, T., Hancock, M., et al. (2014). Cytomegalovirus miRNAs target secretory pathway genes to facilitate formation of the virion assembly compartment and reduce cytokine secretion. *Cell Host Microbe* 15(3)**,** 363-373. doi: 10.1016/j.chom.2014.02.004.

Jheng, J.R., Wang, S.C., Jheng, C.R., and Horng, J.T. (2016). Enterovirus 71 induces dsRNA/PKR-dependent cytoplasmic redistribution of GRP78/BiP to promote viral replication. *Emerg Microbes Infect* 5**,** e23. doi: 10.1038/emi.2016.20.

Ji, W., Chen, J., Mi, Y., Wang, G., Xu, X., and Wang, W. (2016). Platelet-activating factor receptor activation promotes prostate cancer cell growth, invasion and metastasis via ERK1/2 pathway. *Int J Oncol* 49(1)**,** 181-188. doi: 10.3892/ijo.2016.3519.

Jin, H., and Cho, Y. (2017). Structural and functional relationships of FAN1. *DNA Repair (Amst)* 56**,** 135-143. doi: 10.1016/j.dnarep.2017.06.016.

Jinnouchi, H., Morita, K., Tanaka, T., Kajiwara, A., Kawata, Y., Oniki, K., et al. (2015). Interactive effects of a common gamma-glutamyltransferase 1 variant and low high-density lipoprotein-cholesterol on diabetic macro- and micro-angiopathy. *Cardiovasc Diabetol* 14**,** 49. doi: 10.1186/s12933-015-0212-5.

Jones, D.T., Lechertier, T., Reynolds, L.E., Mitter, R., Robinson, S.D., Kirn-Safran, C.B., et al. (2013). Endogenous ribosomal protein L29 (RPL29): a newly identified regulator of angiogenesis in mice. *Dis Model Mech* 6(1)**,** 115-124. doi: 10.1242/dmm.009183.

Katoh, H., Kubota, T., Nakatsu, Y., Tahara, M., Kidokoro, M., and Takeda, M. (2017). Heat Shock Protein 90 Ensures Efficient Mumps Virus Replication by Assisting with Viral Polymerase Complex Formation. *J Virol* 91(6). doi: 10.1128/jvi.02220-16.

Kohda, A., Yamazaki, S., and Sumimoto, H. (2016). The Nuclear Protein IkappaBzeta Forms a Transcriptionally Active Complex with Nuclear Factor-kappaB (NF-kappaB) p50 and the Lcn2 Promoter via the N- and C-terminal Ankyrin Repeat Motifs. *J Biol Chem* 291(39)**,** 20739-20752. doi: 10.1074/jbc.M116.719302.

Kumar, S., Jain, A., Farzam, F., Jia, J., Gu, Y., Choi, S.W., et al. (2018). Mechanism of Stx17 recruitment to autophagosomes via IRGM and mammalian Atg8 proteins. *J Cell Biol* 217(3)**,** 997-1013. doi: 10.1083/jcb.201708039.

Li, C.C., Wu, T.S., Huang, C.F., Jang, L.T., Liu, Y.T., You, S.T., et al. (2012). GTP-binding-defective ARL4D alters mitochondrial morphology and membrane potential. *PLoS One* 7(8)**,** e43552. doi: 10.1371/journal.pone.0043552.

Lin, M.H., Sivakumaran, H., Jones, A., Li, D., Harper, C., Wei, T., et al. (2014). A HIV-1 Tat mutant protein disrupts HIV-1 Rev function by targeting the DEAD-box RNA helicase DDX1. *Retrovirology* 11**,** 121. doi: 10.1186/s12977-014-0121-9.

Liu, O., and Grant, B.D. (2015). Basolateral Endocytic Recycling Requires RAB-10 and AMPH-1 Mediated Recruitment of RAB-5 GAP TBC-2 to Endosomes. *PLoS Genet* 11(9)**,** e1005514. doi: 10.1371/journal.pgen.1005514.

Loaiza, N., Oldoni, F., and Kuivenhoven, J.A. (2017). Novel regulators of plasma lipid levels. *Curr Opin Lipidol* 28(3)**,** 231-240. doi: 10.1097/mol.0000000000000416.

Matsuda, K., Yoshida, K., Taya, Y., Nakamura, K., Nakamura, Y., and Arakawa, H. (2002). p53AIP1 regulates the mitochondrial apoptotic pathway. *Cancer Res* 62(10)**,** 2883-2889.

Mi, Y., Yu, M., Zhang, L., Sun, C., Wei, B., Ding, W., et al. (2016). COPB2 Is Upregulated in Prostate Cancer and Regulates PC-3 Cell Proliferation, Cell Cycle, and Apoptosis. *Arch Med Res* 47(6)**,** 411-418. doi: 10.1016/j.arcmed.2016.09.005.

Mishima, T., Fujiwara, T., Sanada, M., Kofuji, T., Kanai-Azuma, M., and Akagawa, K. (2014). Syntaxin 1B, but not syntaxin 1A, is necessary for the regulation of synaptic vesicle exocytosis and of the readily releasable pool at central synapses. *PLoS One* 9(2)**,** e90004. doi: 10.1371/journal.pone.0090004.

Pan, S., Cheng, X., and Sifers, R.N. (2013). Golgi-situated endoplasmic reticulum alpha-1, 2-mannosidase contributes to the retrieval of ERAD substrates through a direct interaction with gamma-COP. *Mol Biol Cell* 24(8)**,** 1111-1121. doi: 10.1091/mbc.E12-12-0886.

Perri, V., Pellegrino, M., Ceccacci, F., Scipioni, A., Petrini, S., Gianchecchi, E., et al. (2017). Use of short interfering RNA delivered by cationic liposomes to enable efficient down-regulation of PTPN22 gene in human T lymphocytes. *PLoS One* 12(4)**,** e0175784. doi: 10.1371/journal.pone.0175784.

Petrenko, A.A., Pavlova, L.S., Karseladze, A.I., Kisseljov, F.L., and Kisseljova, N.P. (2006). Downregulation of genes encoding for subunits of adaptor complex-3 in cervical carcinomas. *Biochemistry (Mosc)* 71(10)**,** 1153-1160.

Radhakrishnan, V.M., Putnam, C.W., and Martinez, J.D. (2012). Activation of phosphatidylinositol 3-kinase (PI3K) and mitogen-activated protein kinase (MAPK) signaling and the consequent induction of transformation by overexpressed 14-3-3gamma protein require specific amino acids within 14-3-3gamma N-terminal variable region II. *J Biol Chem* 287(52)**,** 43300-43311. doi: 10.1074/jbc.M112.397877.

Reshetnyak, A.V., Murray, P.B., Shi, X., Mo, E.S., Mohanty, J., Tome, F., et al. (2015). Augmentor alpha and beta (FAM150) are ligands of the receptor tyrosine kinases ALK and LTK: Hierarchy and specificity of ligand-receptor interactions. *Proc Natl Acad Sci U S A* 112(52)**,** 15862-15867. doi: 10.1073/pnas.1520099112.

Rogers, A., Nesbit, M.A., Hannan, F.M., Howles, S.A., Gorvin, C.M., Cranston, T., et al. (2014). Mutational analysis of the adaptor protein 2 sigma subunit (AP2S1) gene: search for autosomal dominant hypocalcemia type 3 (ADH3). *J Clin Endocrinol Metab* 99(7)**,** E1300-1305. doi: 10.1210/jc.2013-3909.

Rosa-Ferreira, C., Christis, C., Torres, I.L., and Munro, S. (2015). The small G protein Arl5 contributes to endosome-to-Golgi traffic by aiding the recruitment of the GARP complex to the Golgi. *Biol Open* 4(4)**,** 474-481. doi: 10.1242/bio.201410975.

Schafer, G., Graham, L.M., Lang, D.M., Blumenthal, M.J., Bergant Marusic, M., and Katz, A.A. (2017). Vimentin Modulates Infectious Internalization of Human Papillomavirus 16 Pseudovirions. *J Virol* 91(16). doi: 10.1128/jvi.00307-17.

Shum, M.S., Pasquier, E., Po'uha, S.T., O'Neill, G.M., Chaponnier, C., Gunning, P.W., et al. (2011). gamma-Actin regulates cell migration and modulates the ROCK signaling pathway. *Faseb j* 25(12)**,** 4423-4433. doi: 10.1096/fj.11-185447.

Simpson, J.C., Joggerst, B., Laketa, V., Verissimo, F., Cetin, C., Erfle, H., et al. (2012). Genome-wide RNAi screening identifies human proteins with a regulatory function in the early secretory pathway. *Nat Cell Biol* 14(7)**,** 764-774. doi: 10.1038/ncb2510.

Spalinger, M.R., Lang, S., Gottier, C., Dai, X., Rawlings, D.J., Chan, A.C., et al. (2017). PTPN22 regulates NLRP3-mediated IL1B secretion in an autophagy-dependent manner. *Autophagy* 13(9)**,** 1590-1601. doi: 10.1080/15548627.2017.1341453.

Srisutthisamphan, K., Jirakanwisal, K., Ramphan, S., Tongluan, N., Kuadkitkan, A., and Smith, D.R. (2018). Hsp90 interacts with multiple dengue virus 2 proteins. *Sci Rep* 8(1)**,** 4308. doi: 10.1038/s41598-018-22639-5.

Stewart, A.J., Mukherjee, J., Roberts, S.J., Lester, D., and Farquharson, C. (2005). Identification of a novel class of mammalian phosphoinositol-specific phospholipase C enzymes. *Int J Mol Med* 15(1)**,** 117-121.

Strauss, M., Filman, D.J., Belnap, D.M., Cheng, N., Noel, R.T., and Hogle, J.M. (2015). Nectin-like interactions between poliovirus and its receptor trigger conformational changes associated with cell entry. *J Virol* 89(8)**,** 4143-4157. doi: 10.1128/jvi.03101-14.

Takashima, K., Oshiumi, H., Takaki, H., Matsumoto, M., and Seya, T. (2015). RIOK3-mediated phosphorylation of MDA5 interferes with its assembly and attenuates the innate immune response. *Cell Rep* 11(2)**,** 192-200. doi: 10.1016/j.celrep.2015.03.027.

Tong, S., Xia, T., Fan, K., Jiang, K., Zhai, W., Li, J.S., et al. (2016). 14-3-3zeta promotes lung cancer cell invasion by increasing the Snail protein expression through atypical protein kinase C (aPKC)/NF-kappaB signaling. *Exp Cell Res* 348(1)**,** 1-9. doi: 10.1016/j.yexcr.2016.08.014.

Tuysuz, B., Bilguvar, K., Kocer, N., Yalcinkaya, C., Caglayan, O., Gul, E., et al. (2014). Autosomal recessive spastic tetraplegia caused by AP4M1 and AP4B1 gene mutation: expansion of the facial and neuroimaging features. *Am J Med Genet A* 164a(7)**,** 1677-1685. doi: 10.1002/ajmg.a.36514.

Wang, Y., Foo, L.Y., Guo, K., Gan, B.Q., Zeng, Q., Hong, W., et al. (2006). Syntaxin 9 is enriched in skin hair follicle epithelium and interacts with the epidermal growth factor receptor. *Traffic* 7(2)**,** 216-226. doi: 10.1111/j.1600-0854.2005.00378.x.

Watkin, L.B., Jessen, B., Wiszniewski, W., Vece, T.J., Jan, M., Sha, Y., et al. (2015). COPA mutations impair ER-Golgi transport and cause hereditary autoimmune-mediated lung disease and arthritis. *Nat Genet* 47(6)**,** 654-660. doi: 10.1038/ng.3279.

Weimer, C., Beck, R., Eckert, P., Reckmann, I., Moelleken, J., Brugger, B., et al. (2008). Differential roles of ArfGAP1, ArfGAP2, and ArfGAP3 in COPI trafficking. *J Cell Biol* 183(4)**,** 725-735. doi: 10.1083/jcb.200806140.

Wu, X.S., Lee, S.H., Sheng, J., Zhang, Z., Zhao, W.D., Wang, D., et al. (2016). Actin Is Crucial for All Kinetically Distinguishable Forms of Endocytosis at Synapses. *Neuron* 92(5)**,** 1020-1035. doi: 10.1016/j.neuron.2016.10.014.

Xu, Y., Martin, S., James, D.E., and Hong, W. (2002). GS15 forms a SNARE complex with syntaxin 5, GS28, and Ykt6 and is implicated in traffic in the early cisternae of the Golgi apparatus. *Mol Biol Cell* 13(10)**,** 3493-3507. doi: 10.1091/mbc.e02-01-0004.

Zhang, T., Ji, D., Wang, P., Liang, D., Jin, L., Shi, H., et al. (2018). The atypical protein kinase RIOK3 contributes to glioma cell proliferation/survival, migration/invasion and the AKT/mTOR signaling pathway. *Cancer Lett* 415**,** 151-163. doi: 10.1016/j.canlet.2017.12.010.

Zhang, Z., Hui, E., Chapman, E.R., and Jackson, M.B. (2010). Regulation of exocytosis and fusion pores by synaptotagmin-effector interactions. *Mol Biol Cell* 21(16)**,** 2821-2831. doi: 10.1091/mbc.E10-04-0285.

Zheng, L.Q., Chi, S.M., and Li, C.X. (2017). Rab23's genetic structure, function and related diseases: a review. *Biosci Rep* 37(2). doi: 10.1042/bsr20160410.
